# mEYEstro software: an automatic tool for standardized refractive surgery outcomes reporting

**DOI:** 10.1186/s12886-023-02904-6

**Published:** 2023-04-21

**Authors:** Mathieu Gauvin, Avi Wallerstein

**Affiliations:** 1 Department of Ophthalmology & Visual Sciences, Faculty of Medicine, McGill University, Montreal, QC Canada; 2 LASIK MD, 1250 Rene-Levesque Blvd W, MD Level, Montreal, QC H3B 4W8 Canada

**Keywords:** Refractive surgery, Standard graphs, LASIK, IOL, Cataract

## Abstract

**Background:**

Standardization for reporting medical outcomes enables clinical study comparisons and has a fundamental role in research reproducibility. In this context, we present mEYEstro, a free novel standalone application for automated standardized refractive surgery graphs. mEYEstro can be used for single and multiple group comparisons in corneal and intraocular refractive surgery patients. In less than 30 s and with minimal user manipulation, mEYEstro automatically creates the required journal standard graphs while simultaneously performing valid statistical analyses.

**Results:**

The software produces the following 11 standard graphs; **Efficacy**: 1. Cumulative uncorrected (UDVA) and corrected visual acuity (CDVA), 2. Difference between UDVA and CDVA, **Safety**: 3. Change in line of CDVA, **Accuracy**: 4. Spherical equivalent (SEQ) to intended target, 5. Attempted vs. achieved SEQ, 6. Defocus equivalent (DEQ) accuracy, 7. Refractive astigmatism accuracy, 8. Target-induced astigmatism vs. Surgically-induced astigmatism, 9. Correction index histogram, 10. Angle of error histogram, **Stability**: 11. SEQ stability over time. Percent proportions, means, standard deviations, Cohen's *d* effect sizes, and *p*-values are calculated and displayed on each graph. All graphs can be easily exported as high-resolution TIFF images for figures to use in scientific manuscripts and presentations.

**Conclusions:**

mEYEstro software enables clinicians, surgeons, and researchers, to easily and efficiently analyze refractive surgery outcomes using the standardized methodology required by several peer-reviewed ophthalmology journals.

**Supplementary Information:**

The online version contains supplementary material available at 10.1186/s12886-023-02904-6.

## Background

The standardization of medical outcome reporting simplifies comparisons between clinical studies and enhances reproducibility [1, 2]. Waring proposed the first set of refractive surgery outcomes reporting standards in 1992 [3] incorporating six standard graphs describing accuracy, efficacy, safety, and stability of surgical procedures [4–10]. A new updated set of nine standard graphs was added to cover astigmatism outcomes [1]. A similar set of guidelines was recently published for lens-based refractive surgery [2]. In the Journal of Refractive Surgery (JRS) [6], Journal of Cataract and Refractive Surgery (JCRS) [7], and Cornea [8], these standard graphs are currently required with each submission assessing post-operative outcomes. Additional journals, including Ophthalmology, also recommend using these standard graphs as part of their author guidelines [10]. By following these specifications, results from specific surgical techniques, studies, case reports, or case series are standardized and easily comparable within and between studies [1].

Refractive surgery standard graphs can be made by purchasing web-based or standalone software designed for refractive surgery outcomes analysis or by downloading free macro-enabled Microsoft Excel spreadsheets [1, 2]. Macro-enabled spreadsheets are difficult to use because they require manual data importation, manual formatting, and manual adjustments, which are time-consuming and are prone to user error. More importantly, they do not allow for automated simultaneous analyses of two comparative groups nor for performing automated "paired" and "unpaired" statistical analyses. Consequently, specialized freeware for the rapid and automated production of all standard graphs remains unavailable, limiting their use.

In this context, we introduce mEYEstro, a free standalone software program that automatically performs statistical analysis and produces standardized refractive surgery graphs. By providing high-definition standard graphs, mEYEstro can assist clinicians and researchers in understanding clinical outcomes and presenting them in accordance with current peer-reviewed journal standards for reproducible research in corneal and intra-ocular refractive surgery.

## Implementation

### Software implementation and system requirements

mEYEstro is programmed and compiled in MATLAB R2023a (MathWorks Inc., Natick, MA, USA) using the MATLAB runtime compiler (MathWorks Inc.). mEYEstro is therefore an executable file (*.exe) that can be run as an independent Desktop application. mEYEstro requires the MATLAB runtime compiler (MRC) to be correctly installed on the computer. The MRC installs automatically with the mEYEstro install. mEYEstro has been tested on Windows 10 Home and Professional, with a 64-bit-operating system and both with 1920 × 1080 and 3840 × 2160 screen resolutions. mEYEstro and the demonstration trial datasets are available to download from https://www.lasikmd.com/media/meyestro. A tutorial video is available at this link (https://www.youtube.com/watch?v=NFlRRHx6ZaI) and a tutorial guideline in Supplementary File C.

### Usage

mEYEstro can be used to automate producing all of the standard refractive surgery graphs, as recommended by various ophthalmology journals [10–14]. The tool was developed specifically for academic research and teaching purposes but can also be used by surgeons looking to understand and improve their clinical outcomes. mEYEstro can be used to examine the visual and refractive outcomes of any corneal or intraocular refractive procedure. The corneal procedures include LASIK, PRK, and SMILE as well as collagen crosslinking, incisional keratotomy, intracorneal rings segments, LASEK, etc. The lens-based procedures include cataract surgery, refractive lens exchange, phakic IOL, etc. mEYEstro can also be utilized to study outcomes of procedures used to treat the various refractive surgery complications that exist today, or any other surgical procedure involving the eye [15–24]. The use of mEYEstro is completely free provided that the user cites the current manuscript when using mEYEstro results in publications, presentations, or other public communications.

### Input data format

To automatically generate the figures, mEYEstro reads data files in Microsoft Excel format (e.g., Datafile.xlsx). Excel was used due to its widespread use and simplicity. There are 20 columns, including 15 that are mandatory for proper mEYEstro functioning. The first five columns are 1) preoperative refraction sphere, 2) preoperative refraction cylinder, 3) preoperative refraction axis, 4) preoperative refraction vertex distance, and 5) preoperative corrected distance visual acuity (CDVA). The next four columns are 6) intended postoperative refraction sphere target, 7) intended postoperative refraction cylinder target, 8) intended postoperative refraction axis target, and 9) intended postoperative refraction vertex distance. If the intended postoperative refraction is plano, columns 6, 7 and 8 should be reported as 0, 0, and 0, respectively. The six next columns are 10) postoperative refraction sphere, 11) postoperative refraction cylinder, 12) postoperative refraction axis, 13) postoperative refraction vertex distance, 14) postoperative CDVA and 15) postoperative uncorrected distance visual acuity (UDVA). The last five columns (columns 16 to 20) are optional and allow the user to report the postoperative spherical equivalent (SEQ) at up to five different time points to generate a standard stability graph. Refraction data must be provided in the point decimal format (e.g., -1.50, 0.75) and using the negative cylinder (-ve) nomenclature. The negative cylinder notation was chosen since it is by far the most widespread notation used among refractive surgeons. For calculation mEYEstro will automatically convert the negative cylinder (-ve) to positive notation (+ ve). The UDVA and CDVA data must be provided as the 20/XX Snellen denominator (e.g., 20–1, 15, 20 + 2, 25, 30–1). An example of a representative mEYEstro data file is presented in Supplementary File A. For users that use LogMAR notation in their charting, a LogMAR to Snellen denominator automatic conversion table is included in Supplementary File B. This automatic conversion table can be used as needed to make automatic conversion of LogMAR values to 20/XX Snellen denominator values. The converted value can simply be pasted in a mEYEstro data file. Users can report their refraction data at any vertex distance (12 mm, 10 mm, 0 mm, etc.). mEYEstro will automatically convert the refractive astigmatism, generally measured at a vertex distance of 12 mm, to the corneal plane (0 mm). Data exclusion is at the user’s discretion prior to data importation. Upon entering your data in the mEYEstro datafile, if Excel is automatically converting Snellen denominator like 25–2 to a date “25-Feb”, please instead type ‘25–2 or set the column number format to “Text”.

### Methods and standard reporting

The mEYEstro software adheres to terminology, calculations, and graphical representations originally described by Waring and Reinstein, as well as Editorials by Reinstein et al. [1–3, 5, 6, 9]. All vectorial analyses adhere to terminology, calculations, and graphical representations originally described by Alpins [10, 11, 13, 14]. The efficacy index is calculated as the ratio of postoperative UDVA (converted to decimal format) to the mean preoperative CDVA (converted to decimal format). The safety index is the ratio of postoperative CDVA (converted to decimal format) to mean preoperative CDVA (converted to decimal format). The SEQ was calculated by adding the sum of the sphere power with half of the cylinder power. The defocus equivalent was calculated as the absolute value of the SEQ plus half the absolute value of the cylinder. Negative cylinder (-ve) to positive notation (+ ve) conversion and vertex distance conversions of refraction data adhere to the methodology described by Alpins [10]. Statistical methodologies are presented in the statistical analyses reporting section of the current paper. For additional methodological details, please contact the corresponding author.

### Program workflow

**Fig. 1:**
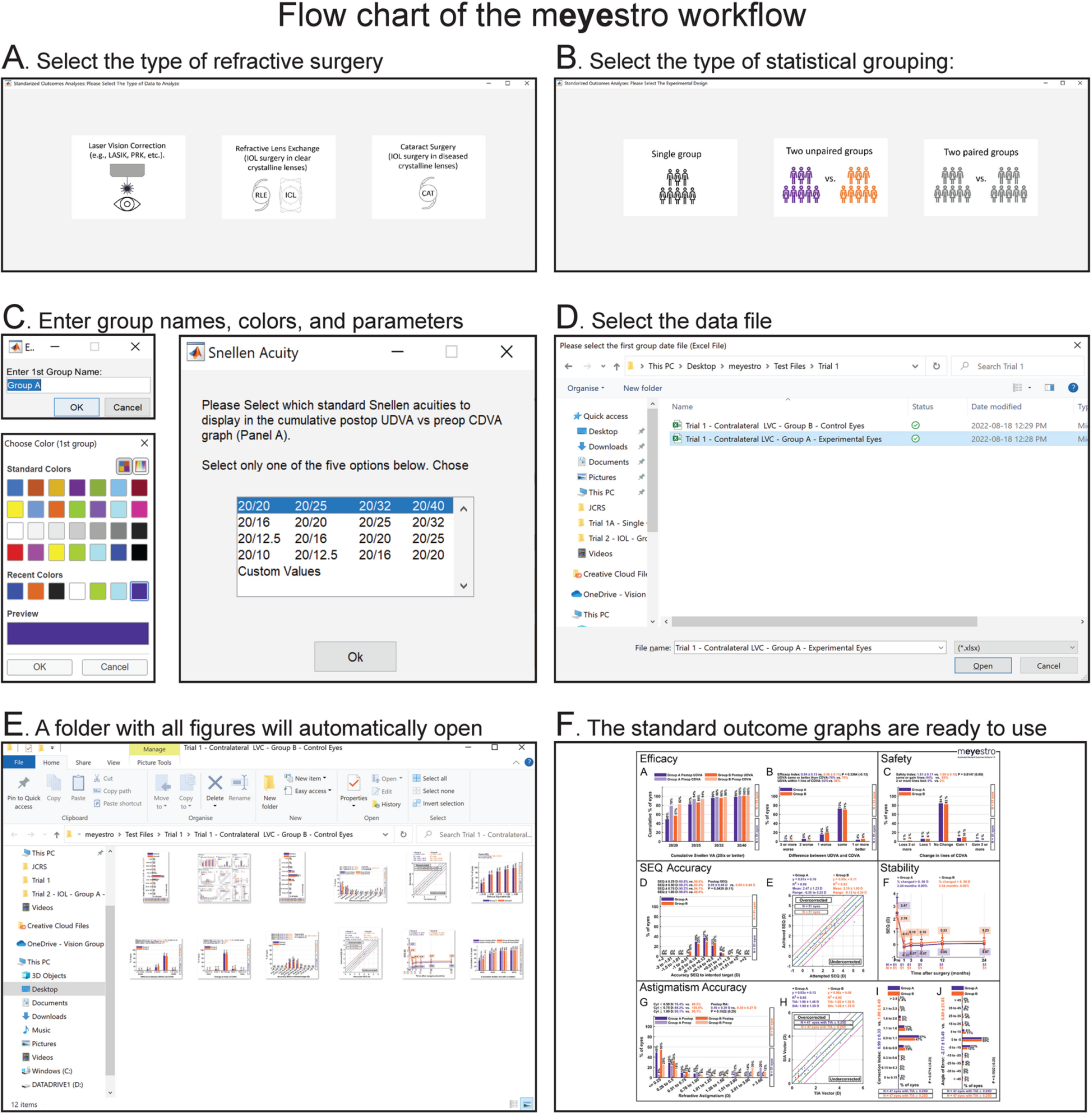
Flow chart of the mEYEstro workflow. **A**-**B** Upon starting the application, the user is invited to select (**A**) the type of refractive surgery and (**B**) the type of statistical grouping. **C** The user is then invited to enter the group(s) name(s), select the colors of the graphs, and parameters. **D** The user is next invited to select the data file. **E** The figures are automatically displayed and saved as high-resolution TIFF images in a folder at the same location as the original Excel data file. This folder automatically opens once the graphs are saved. **F** The user can visualize the generated standard figures

The flow chart of the mEYEstro workflow is shown in Fig. 1. mEYEstro is entirely controlled via a few simple steps and each triggered as the user progresses through the program workflow (Fig. 1). Upon starting the application, the user must choose the type of refractive surgery procedures (LVC, RLE, ICL, CAT) (Fig. 1A), the study design (single group, unpaired groups, paired groups) **(**Fig. 1B), the name of the group(s), the color of the graphs, and the analysis parameters (Snellen lines to display on the UDVA/CDVA graphs, LogMAR threshold for each Snellen optotypes, efficacy & safety index levels, etc.) (Fig. 1C). If the user wants to include a stability graph, additional choices are presented (number of time points, selection of time points to compare, etc.). Finally, the user is invited to select the Excel data file for each group (Fig. 1D). The selected graphs are then generated and automatically saved in a folder as high resolution 400 dpi TIFF images (Fig. 1E). These individual images are ideal for PowerPoint presentations and scientific articles. In addition to the 400 dpi TIFF images, the one-page figure with all 10 standard graphs (Fig. 1F) is exported as an ultra-high definition 1200 dpi TIFF image for journals with higher image quality criteria, such as the Journal of Cataract & Refractive Surgery.

### Efficacy reporting

**Fig. 2:**
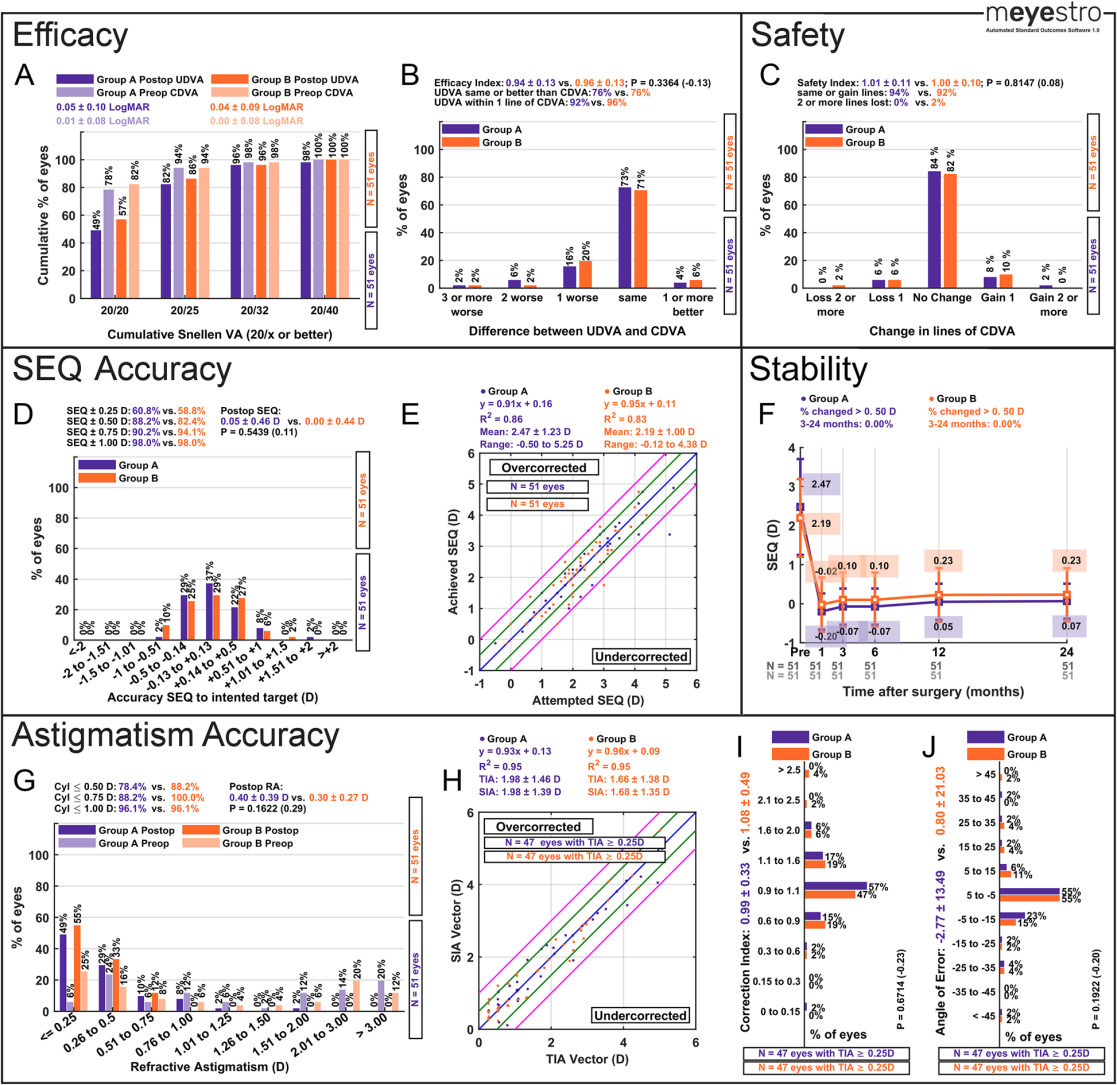
Graphs that are automatically generated by mEYEstro from the provided Trial 1 dataset. The first simulated trial dataset (Trial 1) includes two Excel files (Group A and Group B) and investigates the outcomes of a laser vision correction contralateral eye study comparing two excimer lasers in hyperopic eyes with astigmatism. To generate this standard figure, please use the provided tutorial guideline in Supplementary File C. A tutorial video is available at this link (https://www.youtube.com/watch?v=NFlRRHx6ZaI)

**Fig. 3:**
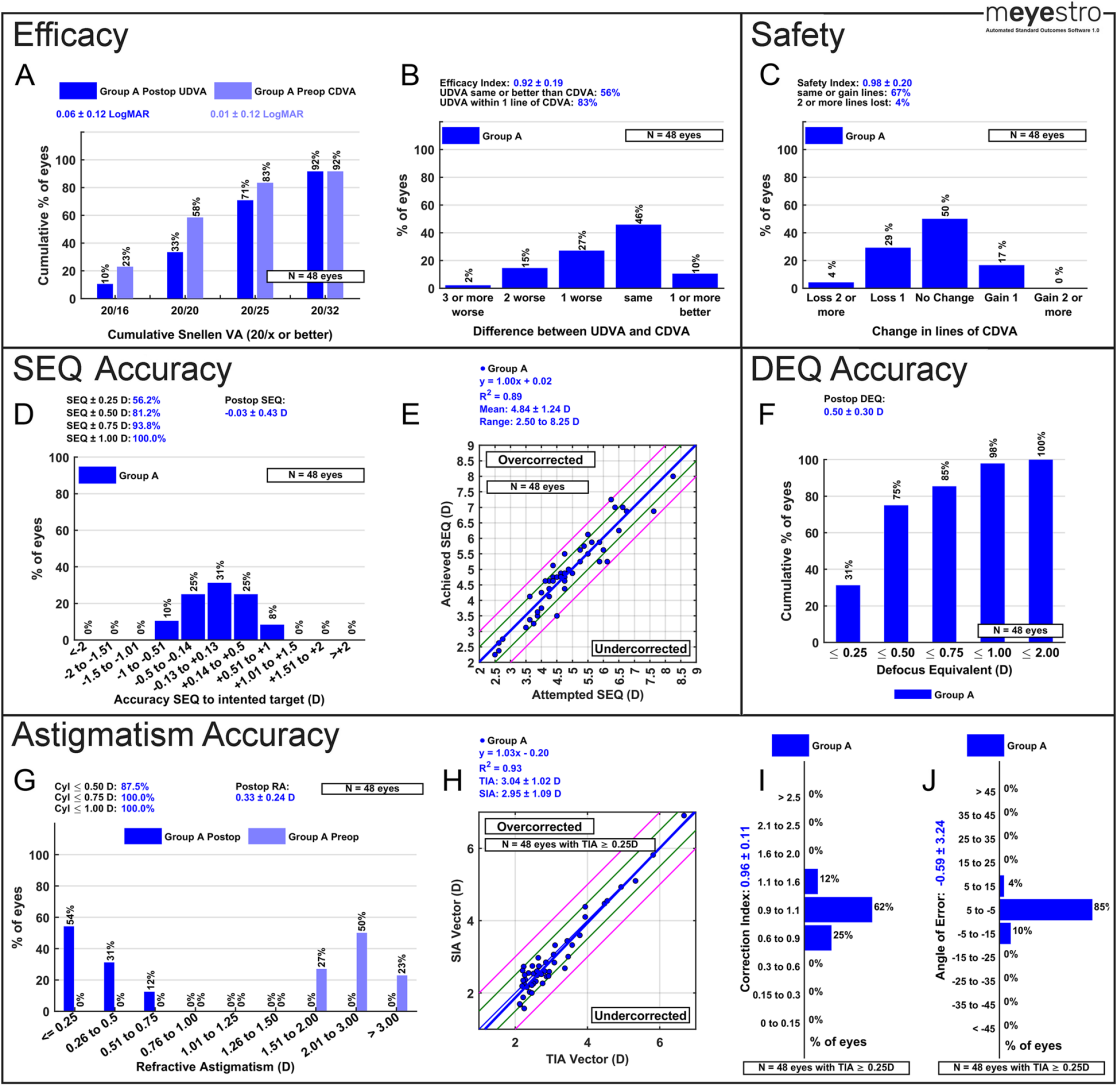
Graphs that are automatically generated by mEYEstro from the provided Trial 2 dataset. The second dataset (Trial 2) is comprised of simulated data from a single group in order to investigate the outcomes of a toric Phakic IOL (PIOL) in hyperopic eyes with moderate to high astigmatism. To generate this standard figure, please use the provided tutorial guideline in Supplementary File C. A tutorial video is available at this link (https://www.youtube.com/watch?v=NFlRRHx6ZaI)

**Fig. 4:**
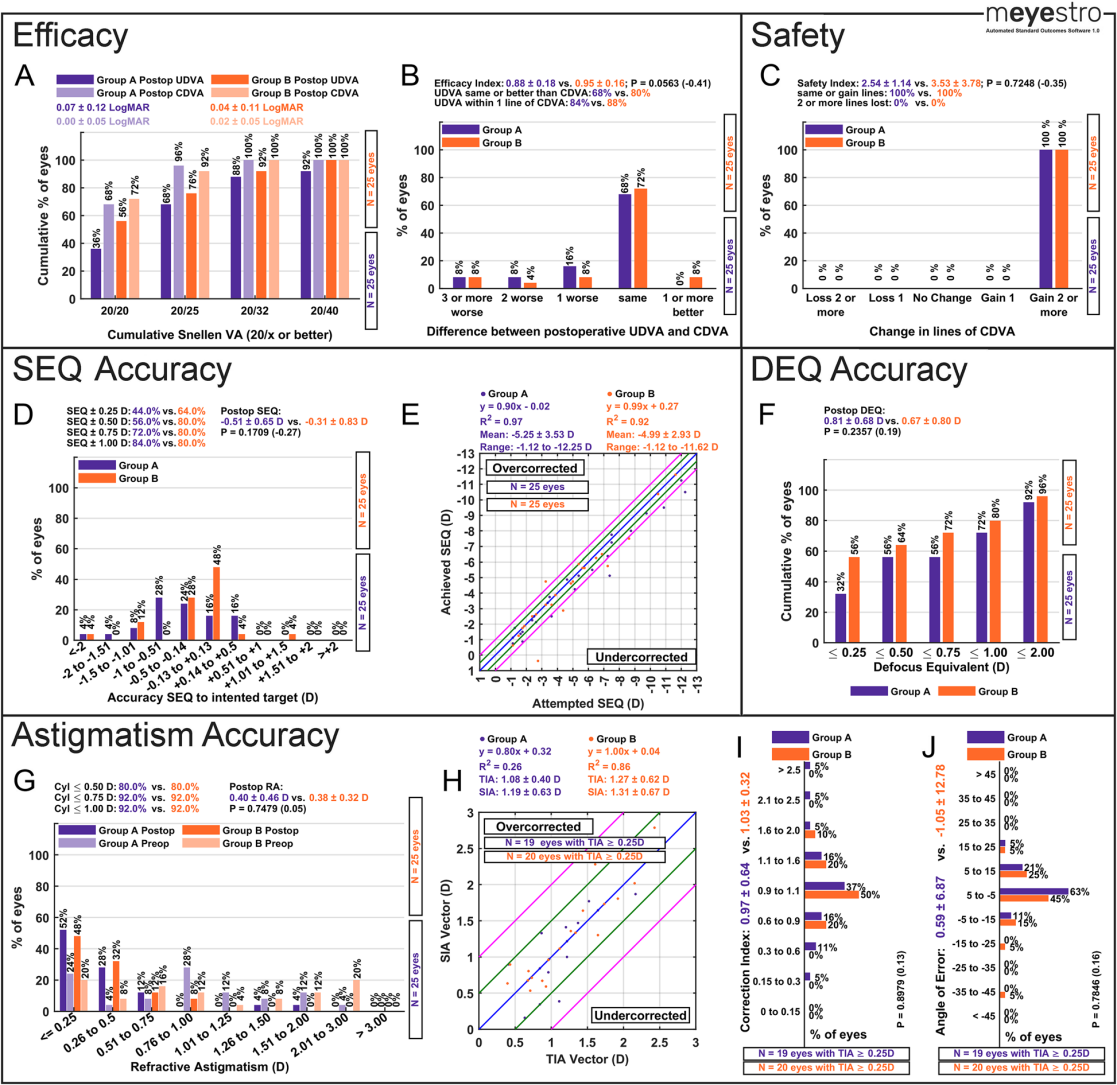
Graphs that are automatically generated by mEYEstro from the provided Trial 3 dataset. The third simulated dataset (Trial 3) includes two files (Group A and Group B), used to investigate the outcomes of two cataract surgery groups, comparing two biometers, in myopic-astigmatism eyes. To generate this standard figure, please use the provided tutorial guideline in Supplementary File C. A tutorial video is available at this link (https://www.youtube.com/watch?v=NFlRRHx6ZaI)

Efficacy analyses include the preoperative and postoperative cumulative Snellen uncorrected (UDVA) and corrected visual acuity (CDVA) graph (Panel A in Figs. 2, 3 snd 4) and the difference between postop UDVA and preop CDVA graph (Panel B in Figs. 2, 3 and 4). These two graphs allow the user to visualize and report standard visual outcomes. Panel A also includes the average (± standard deviations) of the preoperative and postoperative UDVA and CDVA in LogMAR values. The Panel B graph also reports the average efficacy index. The number of eyes per group is also displayed in Panel B. If two groups are analyzed, the *p*-value and effect size between groups is also displayed. For cataract surgery, the postoperative UDVA is compared to postoperative CDVA instead of preoperative CDVA, in agreement with current journal standards.

### Safety reporting

Safety analyses include the change in lines of CDVA (Panel C in Figs. 2, 3 and 4). This graph allows the user to visualize procedures safety in terms of corrected visual acuity line gain and line loss from preop to postop. The graph also reports the average safety index. The number of eyes per group is also displayed in Panel C. If two groups are analyzed, the *p*-value and effect size between groups is also displayed.

### Spherical equivalent accuracy reporting

SEQ accuracy analyses include the accuracy of SEQ to intended target histogram (Panel D in Figs. 2, 3 and 4) and the achieved SEQ vs attempted SEQ scattergram (Panel E in Figs. 2, 3 and 4). These two graphs allow the user to visualize accuracy outcomes. Panel D displays the percentage of eyes within 0.25, 0.50, 0.75 and 1.00 D of intended target, as well as the average postop SEQ to intended target. The number of eyes per group is also displayed in Panel D. If two groups are analyzed, the *p*-value and the effect size between groups is also displayed. Panel E also displays the linear regression equation, the R^2^, the average attempted SEQ, and the range of attempted SEQ.

### Stability reporting

For longitudinal studies where stability over time is an important part of the analyses, a standard SEQ stability graph is required. This graph shows the preoperative SEQ and the postoperative SEQ at up to 5 time points for each group. For example, the user can provide the 1, 3, 6, 12, and 24 months SEQ data in the 5 last columns of the data file and mEYEstro will generate the graph (Panel F in Fig. 2) from the provided data, automatically calculating the mean SEQ at each time point. mEYEstro will also calculate the percentage of eyes with a SEQ change greater than ± 0.50 D between two selected time points. For example, between the 3 months and 24 months postop. Since not all research questions require a longitudinal analysis, the stability analyses are optional and the user may leave the last five columns in the data file blank. The number of eyes at each time point and each group is also displayed at the bottom of Panel F.

### Defocus equivalent accuracy reporting

When the user does not include a stability graph, a DEQ accuracy graph will automatically be included instead. Defocus equivalent (DEQ) accuracy analyses include the postoperative DEQ histogram (Panel F in Figs. 3 and 4). By default, this graph shows the percentage of eyes with a DEQ within 0.25, 0.50, 0.75, 1.00 and 2.00 D. The average postoperative DEQ and the number of eyes per group are also displayed in Panel F. If two groups are analyzed, the *p*-value and the effect size between groups is also displayed.

### Astigmatism accuracy and vector reporting

Standard astigmatism analyses include the postoperative refractive astigmatism graph (Panel G in Figs. 2, 3 and 4), the target-induced astigmatism (TIA) vector vs surgically-induced astigmatism (SIA) vector scattergram (Panel H in Figs. 2, 3 and 4), the Correction Index histogram (Panel I in Figs. 2, 3 and 4), and the Angle of Error histogram (Panel J in Figs. 2, 3 and 4). Panel G display the percentage of eyes within 0.50, 0.75 and 1.00 D of plano postoperative astigmatism, as well as the average postop refractive astigmatism. Panel H also displays the linear regression equation, the R^2^, the average TIA and SIA, and the range of attempted SEQ. The number of eyes per group is also displayed in Panels G, H, I and J. If two groups are analyzed, the *p*-value and the effect size between groups is also displayed. For more detailed formulas and calculations, the interested reader can consult previous literature [10–14]. For advanced standard vector graphs, we have described and provided AstigMATIC tool, available at www.lasikmd.com/media/astigmatic.

### Statistical analyses reporting

When comparing two groups, the mEYEstro software automatically selects and uses the appropriate statistical hypothesis tests. The Kolmogorov–Smirnov test is first used to test if the preoperative and postoperative variables are normally distributed. Unpaired sample T-tests and non-parametric Mann Whitney U-tests are then used where applicable to compared outcomes between two independent groups. Paired samples T-tests or non-parametric Wilcoxon signed-rank test tests are used where applicable to compare two paired groups. Statistical significance is set at p < 0.05 and all data are reported as means ± standard deviations (SD). Effect size (ES), expressed as the Cohen’s *d* is also automatically calculated to better quantify the differences between groups. The effect size is an important indicator of clinical significance. For interpretation, we recommend the user follow the Cohen criteria, where *d* < 0.20 is considered as non-clinically relevant. For greater statistical validity, the user should include the outcome from one eye per patient in the input data file, such as the dominant eye or a randomly selected eye. mEYEstro currently has no feature for comparisons of 3 of more groups using ANOVA.

## Results and discussion

A total of 3 simulated refractive surgery datasets were produced to test and demonstrate the capabilities and all features of mEYEstro. The first simulated trial dataset (Trial 1) includes two Excel files (Group A and Group B) and investigates the outcomes of a laser vision correction contralateral eye study comparing two excimer lasers in hyperopic eyes with astigmatism. The second dataset (Trial 2) comprised simulated data from a single group in order to investigate the outcomes of a toric Phakic IOL (PIOL) in hyperopic eyes with moderate to high astigmatism. The third simulated dataset (Trial 3) included two files (Group A and Group B), in order to investigate the outcomes of two cataract surgery groups, comparing two biometers, in myopic-astigmatism eyes. In each case, mEYEstro was used to read the datasets (Excel files) and to automatically generate all the standard graphs from the provided data, as shown in Figs. 2, 3 and 4. The interested reader can reproduce those graphs using mEYEstro on their own computer with the 3 provided trial datasets. A tutorial video is available at this link (https://www.youtube.com/watch?v=NFlRRHx6ZaI) and a tutorial guideline in Supplementary File C.

One limitation of mEYEstro is that the user cannot modify or fully customize mEYEstro graphs and features. We chose the executable (*.exe) format to prevent users from modifying or copying the source code, which could then lead to a lack a standardization over time. We elected to fix the format of the mEYEstro graphs so that they would be in accordance with current journal standards [1, 25]. The latter will facilitate comparisons between studies. mEYEstro is currently limited to the 11 standard refractive surgery graphs discussed in this article. While these figures cover the main outcome measures for refractive surgery, supplementary vectorial astigmatism analyses are also recommended [1, 2]. For advanced vector analysis graphs, the user can download and use our free AstigMATIC tool (available at www.lasikmd.com/media/astigmatic) [26].

### Future additions and improvements to the mEYEstro software

mEYEstro will be updated annually or as needed to avoid obsolescence. Future updates to mEYEstro may include: 1) Advanced enhancement analyses. Note that mEYEstro can already be used to report enhancement outcomes using standard graphs but it does not provide additional non-standardized enhancement analyses at present. Pre-enhancement data can be entered as preoperative refractions and post-enhancement data as postoperative refractions, and mEYEstro will display graphs of enhancement outcomes. 2) Advanced nomogram analyses. In the interim, there is currently a scattergram for attempted versus achieved SEQ correction (Panel E), and another for attempted versus achieved astigmatism correction (Panel H), both of which employ linear regression coefficients. Surgeons can use this to make a basic nomogram and improve their surgical outcomes. 3) Direct LogMAR data entry. In the meantime, our LogMAR to Snellen converter can be used to enter data in LogMAR. 4) Snellen data entry in metric format. For now, online tools and tables can help users convert visual acuity in any format, including Snellen in metric units. 5) Multiple three or more groups analyses with automated ANOVA statistics. In the interim, when comparing 3 or more groups, the interested user can generate single group outcome graphs individually for each group. They can then use their own calculated averages, standard deviations, and sample sizes to derive their own hypothesis tests, including ANOVAs. Users who have additional suggestions to make are encouraged to contact us.

### Significance of the mEYEstro software

Refractive surgery analyses are extensive and subtle nuances cannot be fully captured in a single graphical display [10–14]. The mEYEstro automated outcomes software provides a simple approach whereby all graphs are used to answer distinct questions about the efficacy, safety, accuracy, and stability of the procedure. Such analysis enables the cause of an inaccurate surgical correction to be understood and the effectiveness of a treatment to be fully evaluated [10–14]. Many authors, research presenters and clinicians are not able to perform accurate analyses in their studies since a free specialized software for standardized refractive surgery graphs and statistical analyses is unavailable. We therefore developed mEYEstro to meet their needs. It is a fully automated and easy-to-use freeware, designed to analyze outcomes of any refractive procedure creating an output as per the latest standards prescribed by JRS [1], JCRS [25], and Cornea [8]. Note that there are alternate paid software options, including ASSORT, SurgiVision DataLink, Datagraph-med, or IBRA which have nomograms and surgical planning tools. Future studies might compare those alternative outcomes reporting tools.

## Conclusions

With mEYEstro, we provide a freely downloadable tool for automated detailed reporting of refractive surgery outcomes that can be used by clinicians, surgeons, and researchers to easily display standardized graphs for publication, presentation or clinical knowledge.

## Supplementary Information

**Additional file 1.**

**Additional file 2.**

**Additional file 3.**

## Data Availability

Data reported in the current study are included in this published article and are available in a data repository at http://www.lasikmd.com/media/meyestro.

## Abbreviations

AE: Angle of Error
CDVA: Corrected Distance Visual Acuity
CI: Correction Index
IOL: Intra-Ocular Lens
PIOL: Phakic Intra-Ocular Lens
JCRS: Journal of Cataract and Refractive Surgery
JRS: Journal of Refractive Surgery
LASEK: Laser-Assisted Subepithelial Keratomileusis
LASIK: Laser-Assisted in Situ Keratomileusis
LRS: Laser Refractive Surgery
MRC: MATLAB Runtime Compiler
PRK: Photorefractive Keratectomy
SIA: Surgically-Induced Astigmatism
SMILE: Small Incision Lenticule Extraction
TIA: Target-Induced Astigmatism
UDVA: Uncorrected Distance Visual Acuity
