# Supplementary material for: mEYEstro software: an automatic tool for standardized refractive surgery outcomes reporting
Source: BMC Ophthalmol. 2023 Apr 21;23:171. doi: 10.1186/s12886-023-02904-6 (PMC10120175; doi:10.1186/s12886-023-02904-6)
Supplement: Supplementary file 3 — Additional file 3. [file 12886_2023_2904_MOESM3_ESM.docx]

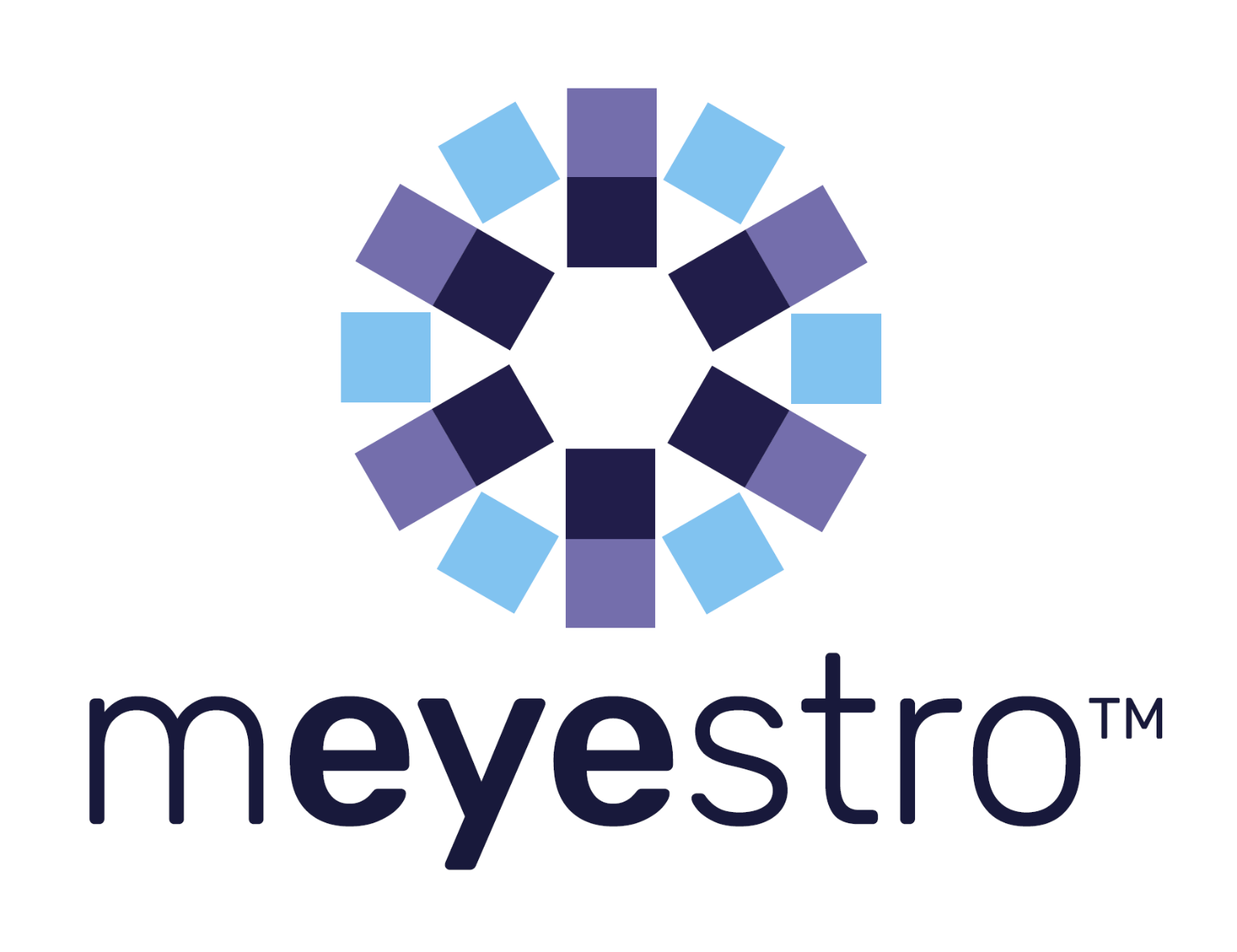


Automated Standard Outcomes Software

**Instruction Manual**

Mathieu Gauvin & Avi Wallerstein ©

**Table of content**

[**Downloading meyestro** 3](#_Toc128656083)

[**Installing meyestro** 4](#_Toc128656084)

[**Using meyestro** 8](#_Toc128656085)

[**Demonstration using the 3 provided Trial Datasets:** 10](#_Toc128656086)

[**Trial 1** 10](#_Toc128656087)

[**Trial 2** 19](#_Toc128656088)

[**Trial 3** 25](#_Toc128656089)

[**Optimal customizations** 32](#_Toc128656090)

[**Displaying custom Snellen lines in Panel A** 32](#_Toc128656091)

[**Customizing or creating your own figure layout** 33](#_Toc128656092)

[**Future customizations** 35](#_Toc128656093)

[**Optional LogMAR to Snellen denominator (20/XX) conversion** 35](#_Toc128656094)

[**What to do if you experiment a bug or crash while using meyestro** 35](#_Toc128656095)

[**Revision notes** 35](#_Toc128656096)

[**Version 1.8** 35](#_Toc128656097)

Dear user, this instruction manual will help you getting started with mEYEstro. The version of mEYEstro that you will be downloading is more recent (1.8 and above) than the one presented in this manual (1.7). Please see the revision notes at the end of this document for details. Thank you for using mEYEstro.

# **Downloading meyestro**

Go on our website [www.lasikmd.com/meyestro.com](http://www.lasikmd.com/meyestro.com) (1), click on “I agree” (2), and then click on the “Download ZIP” button (3), the file will be saved in your “Download” folder (4):


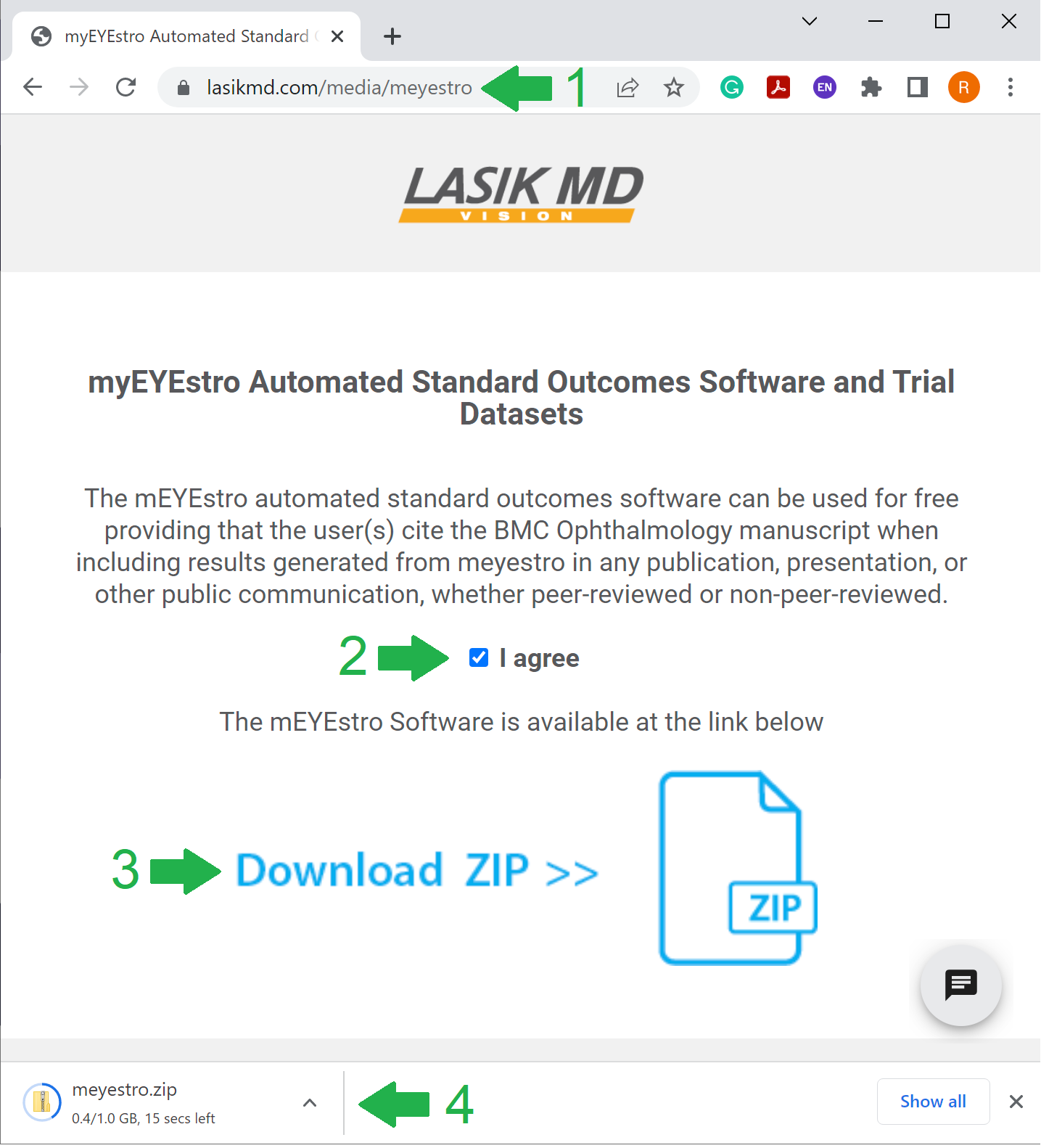


# **Installing meyestro**

Move the downloaded meyestro.zip folder on your Desktop. Right-click on the Zipped folder, and extract the folder to your Desktop (1):


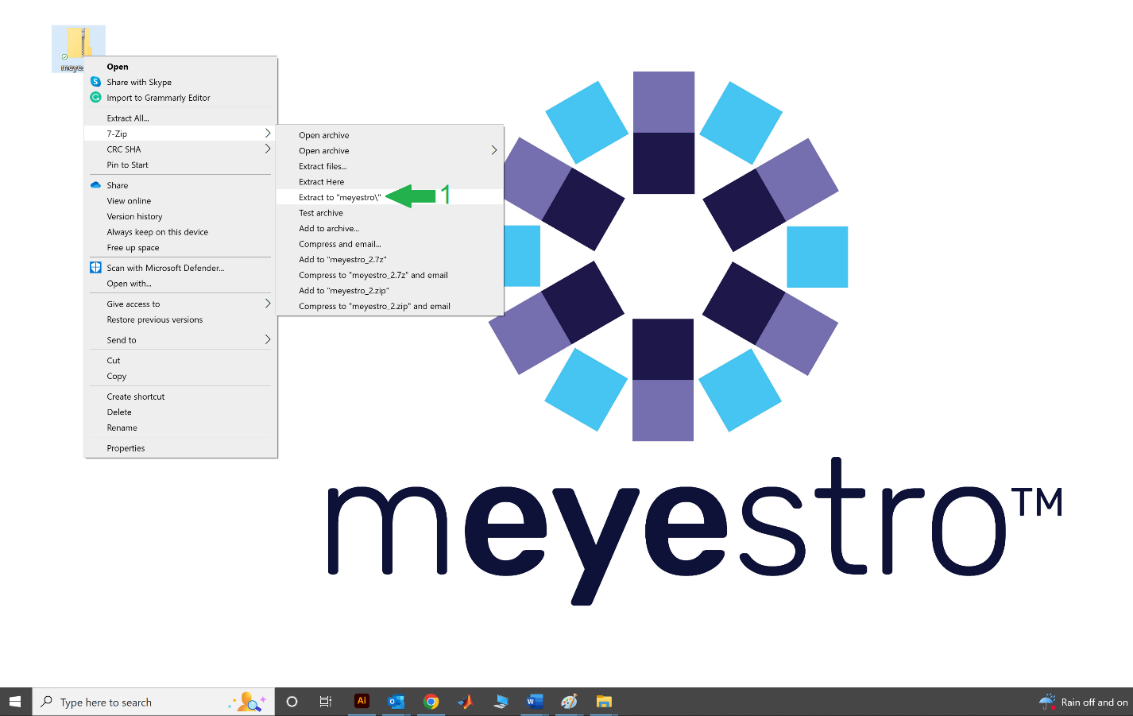


Open the uncompressed meyestro folder (1). This folder contains the Read Me file, which we will please invite you to read before installing meyestro. Once ready to install, double-click on **meyestro_mcr_v1_8.exe** to start the installation process (2):


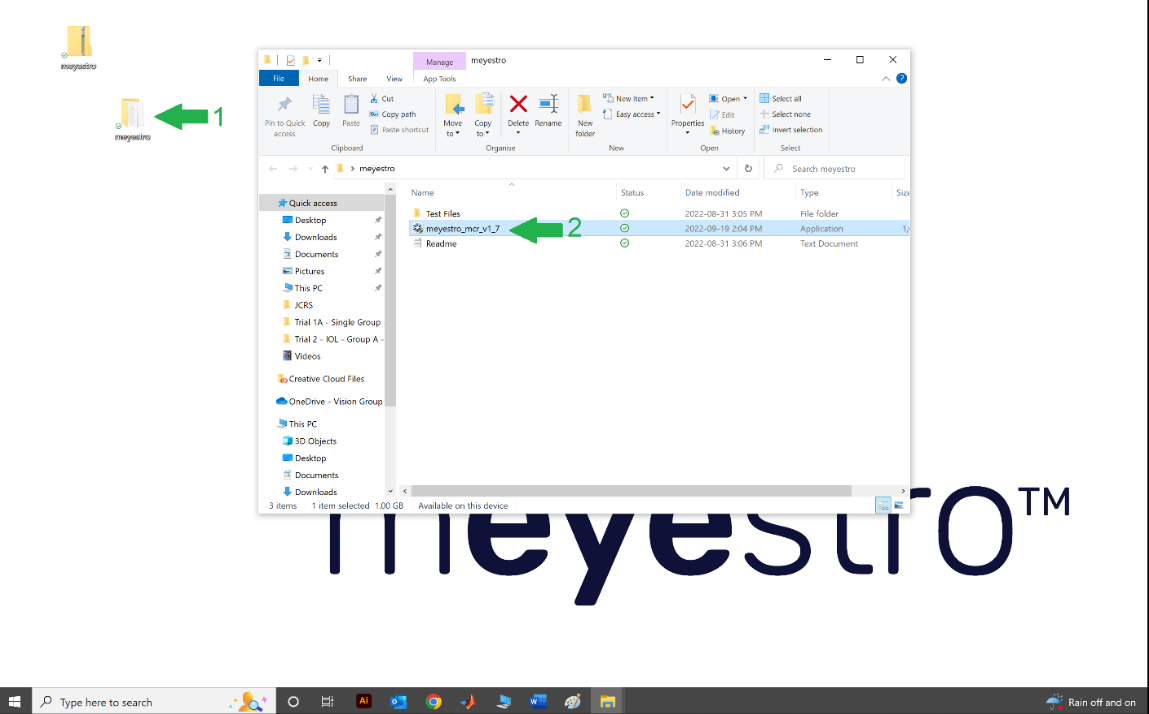


The installer will then start. Click on “Next” (1):


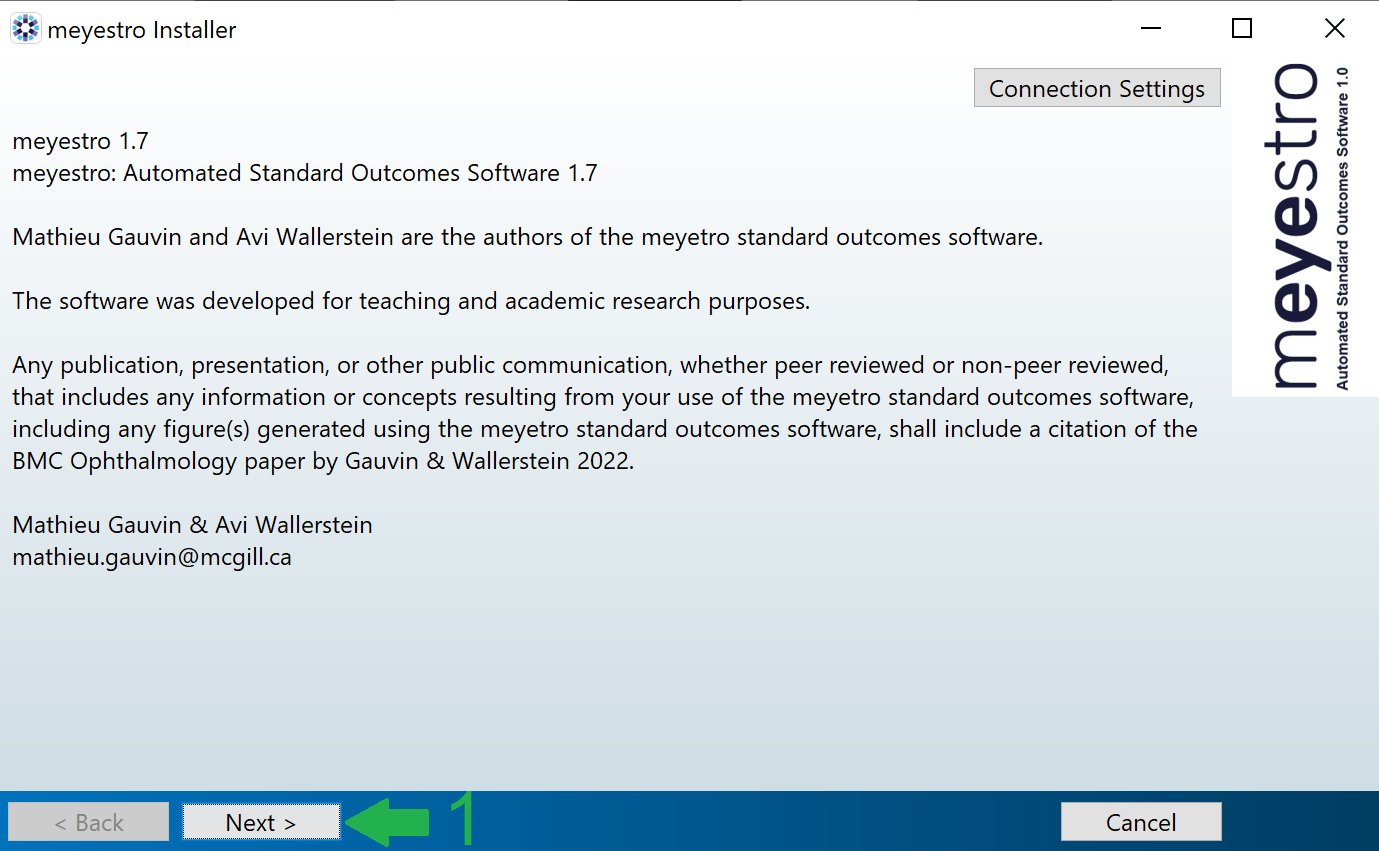


Click on “Add a shortcut to the desktop” (1), and then click on “Next” (2):


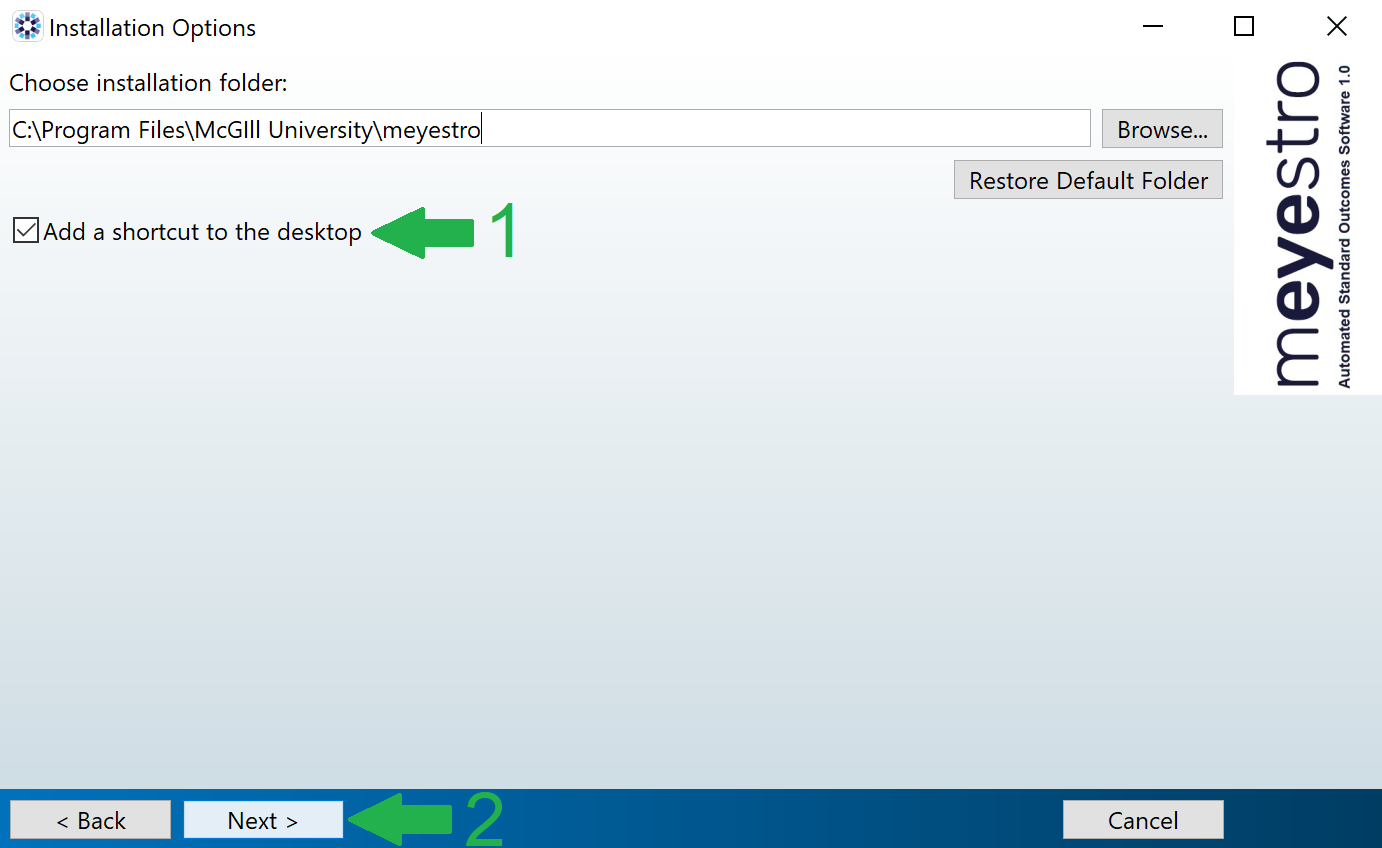


meyestro will then install the Matlab Runtime Compiler required to run meyestro. Click “Next” (1):


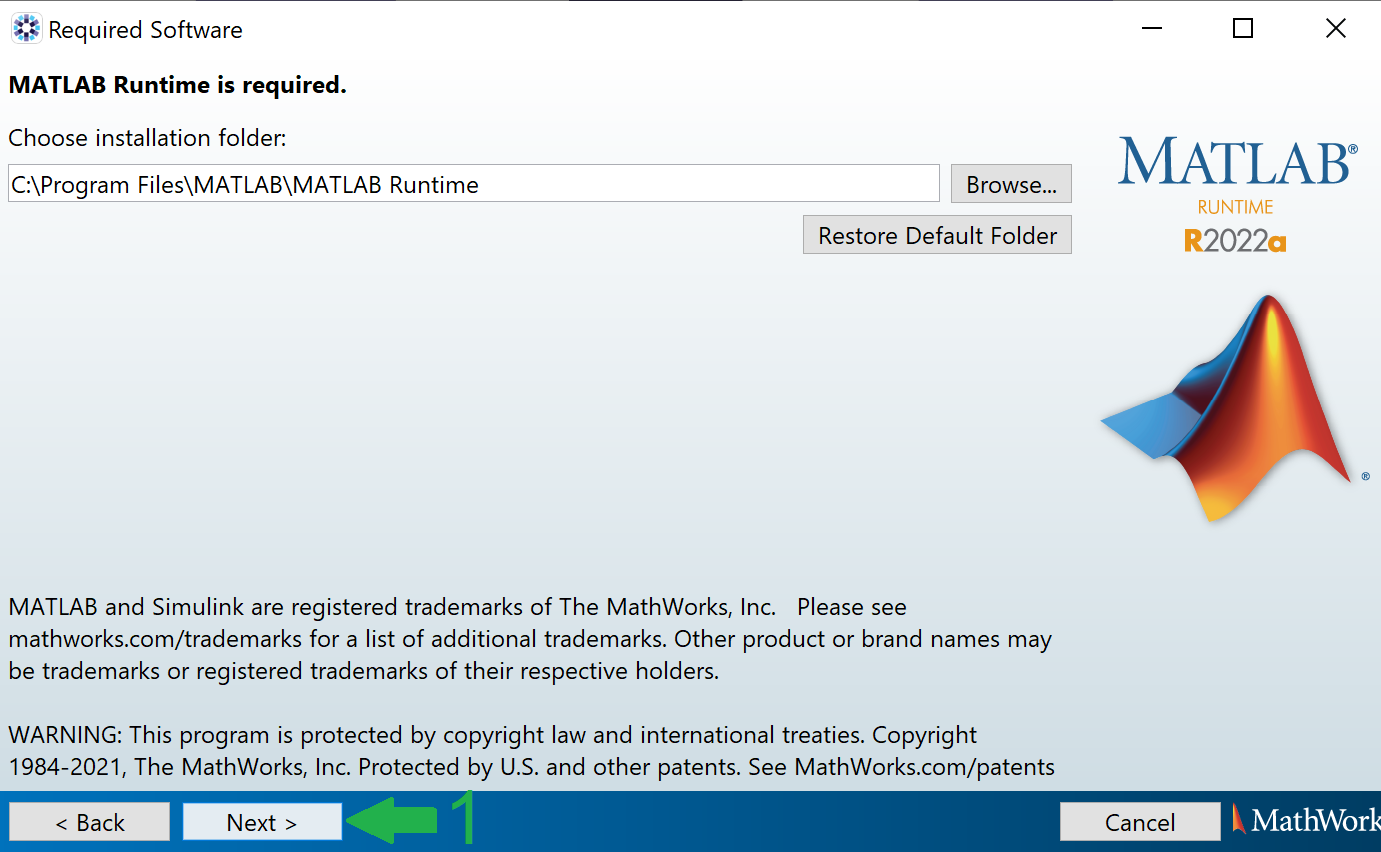


Click on “yes” to accept the license agreement (1), and then click on “Next” (2):


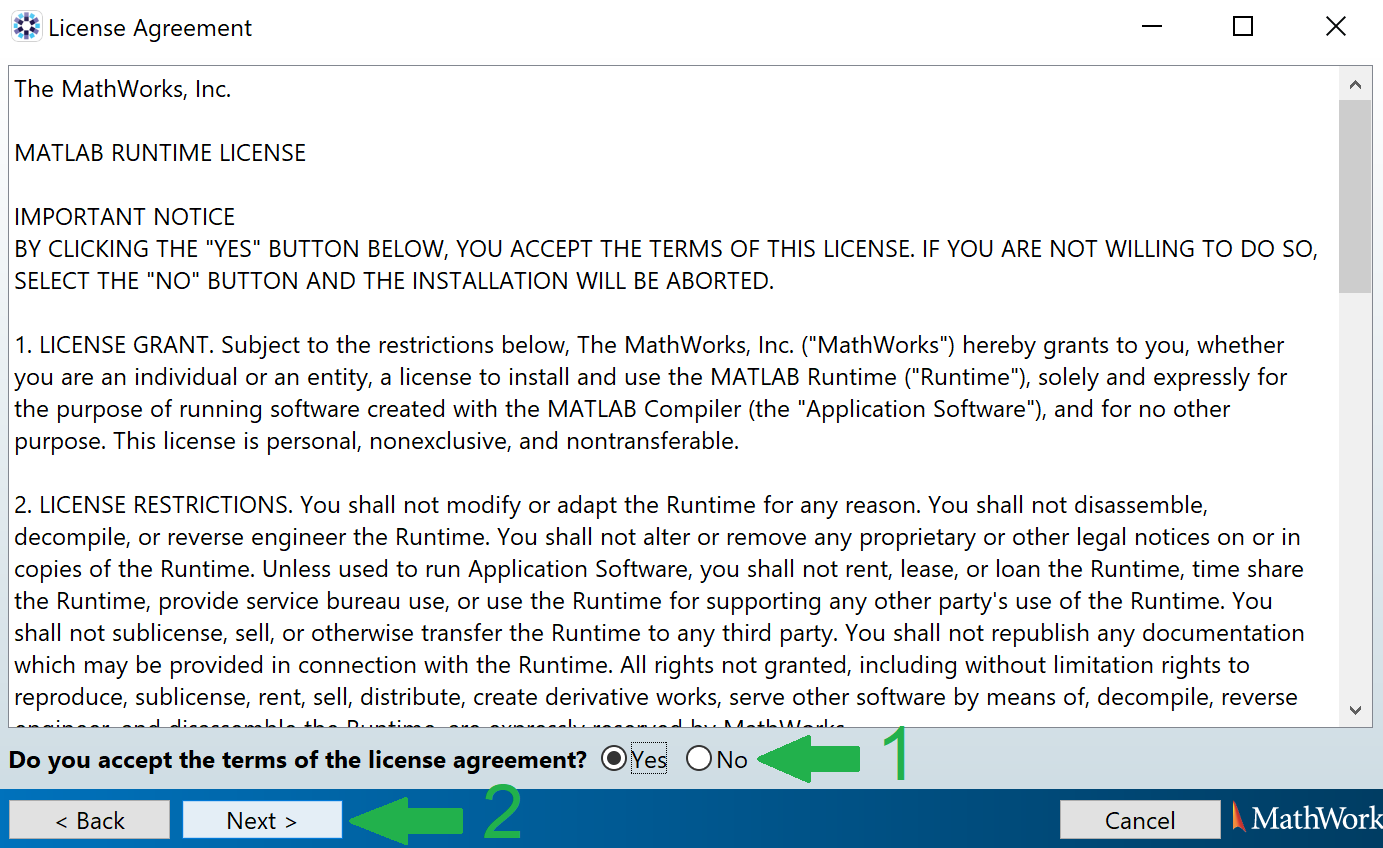


Click on install (1) to confirm and start the installation:


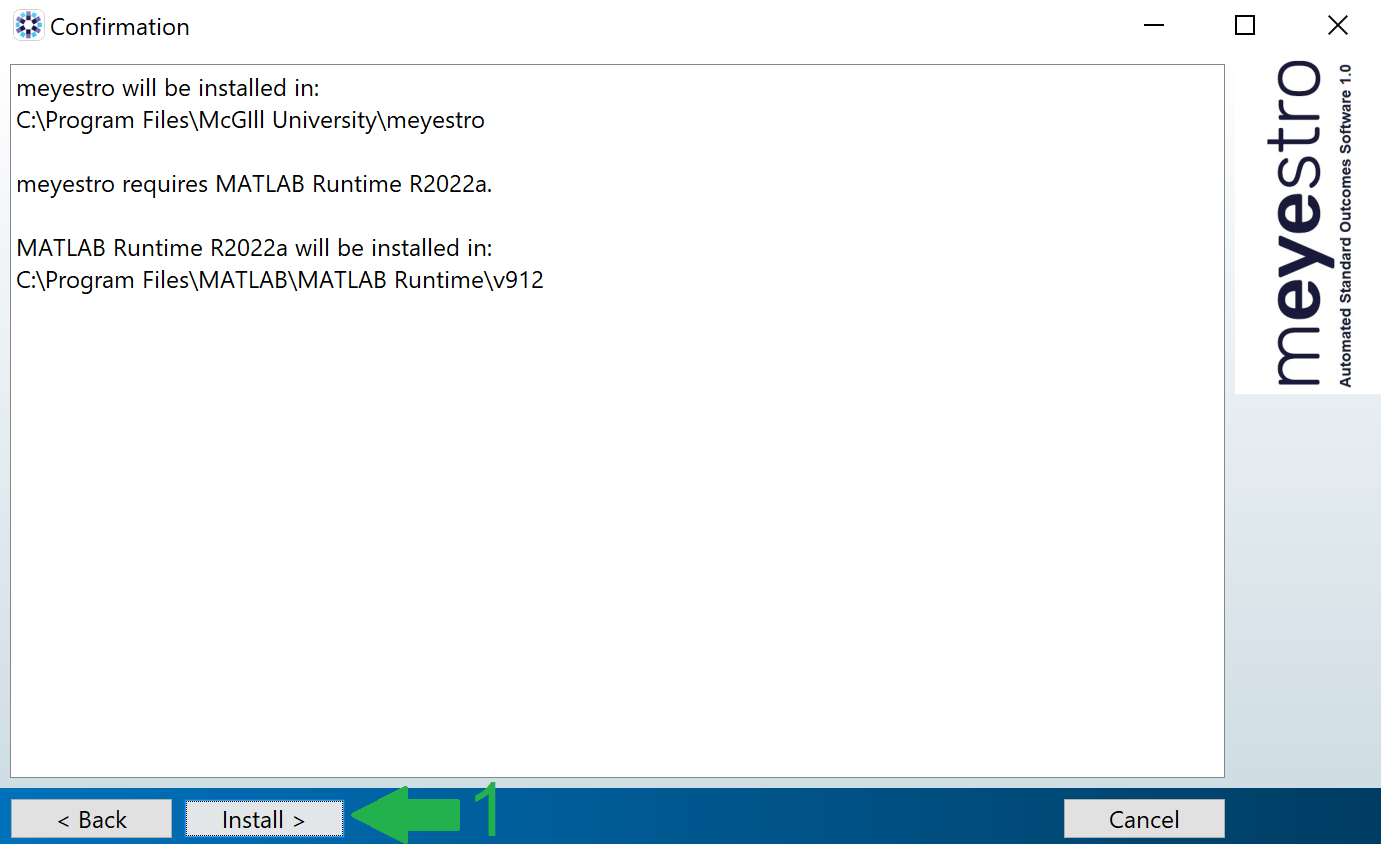


The installation starts:


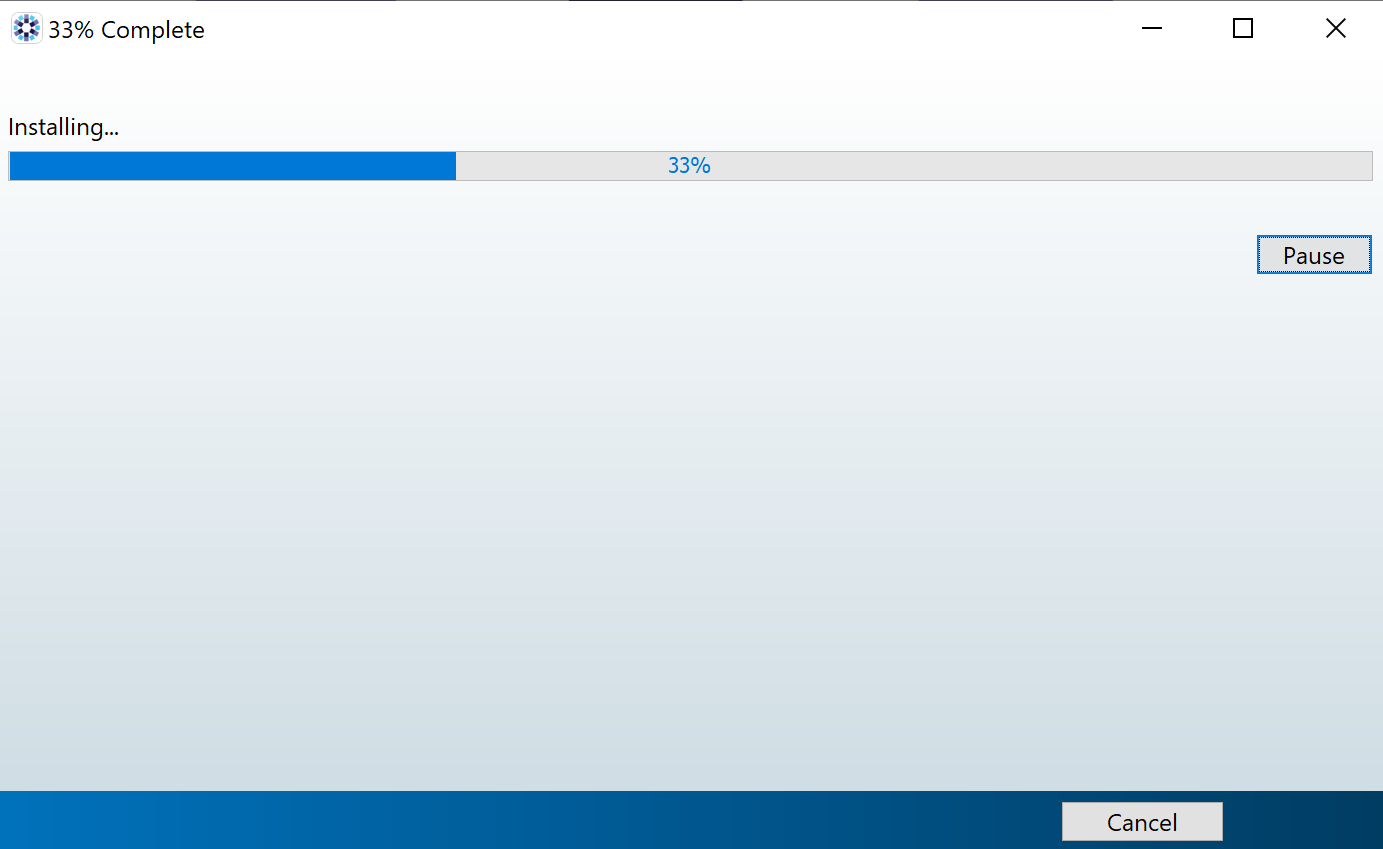


Once the installation is completed successfully, please click on “Finish” (1):


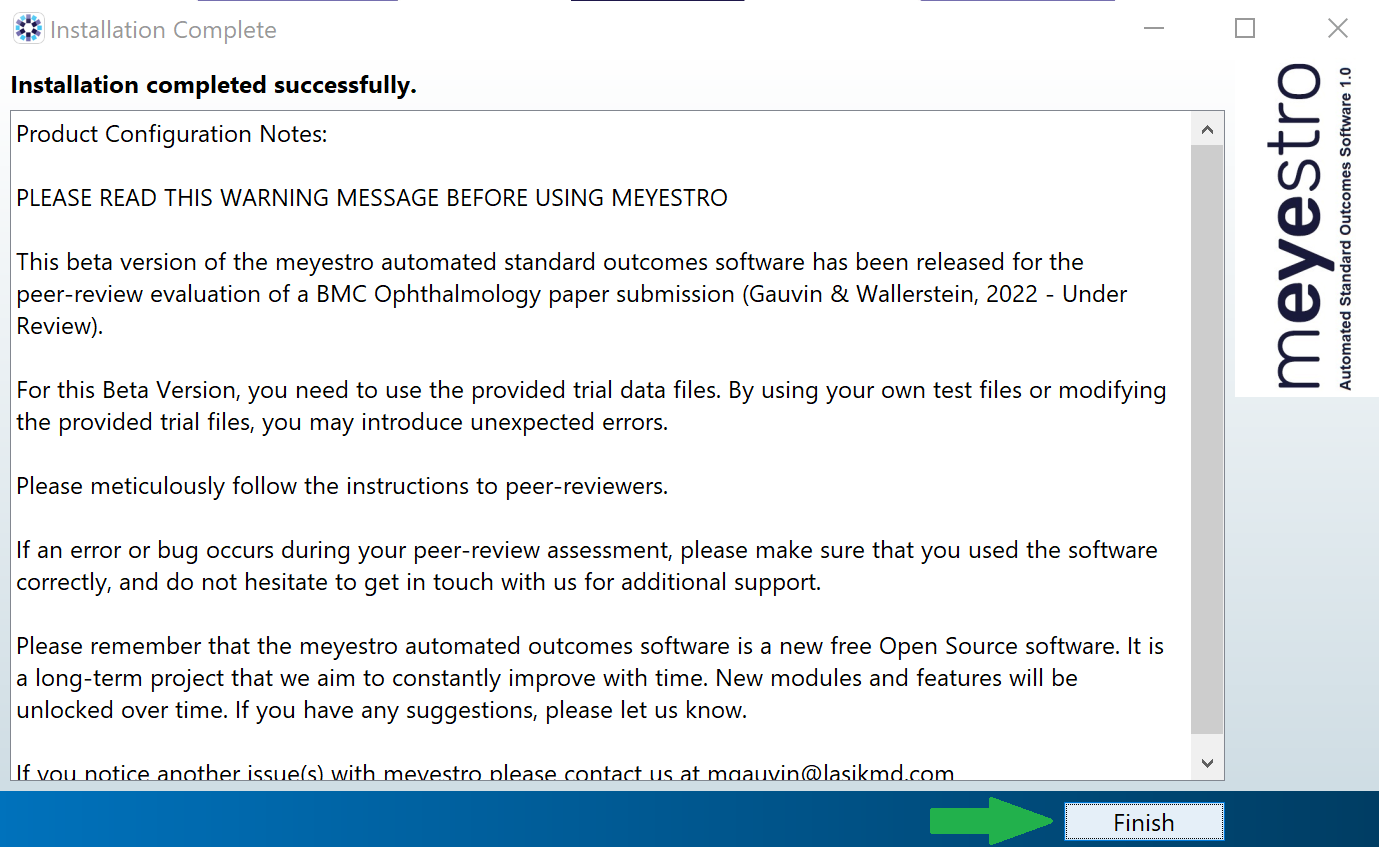


# **Using meyestro**

To start meyestro, double-click on the meyestro application on your Desktop (1):


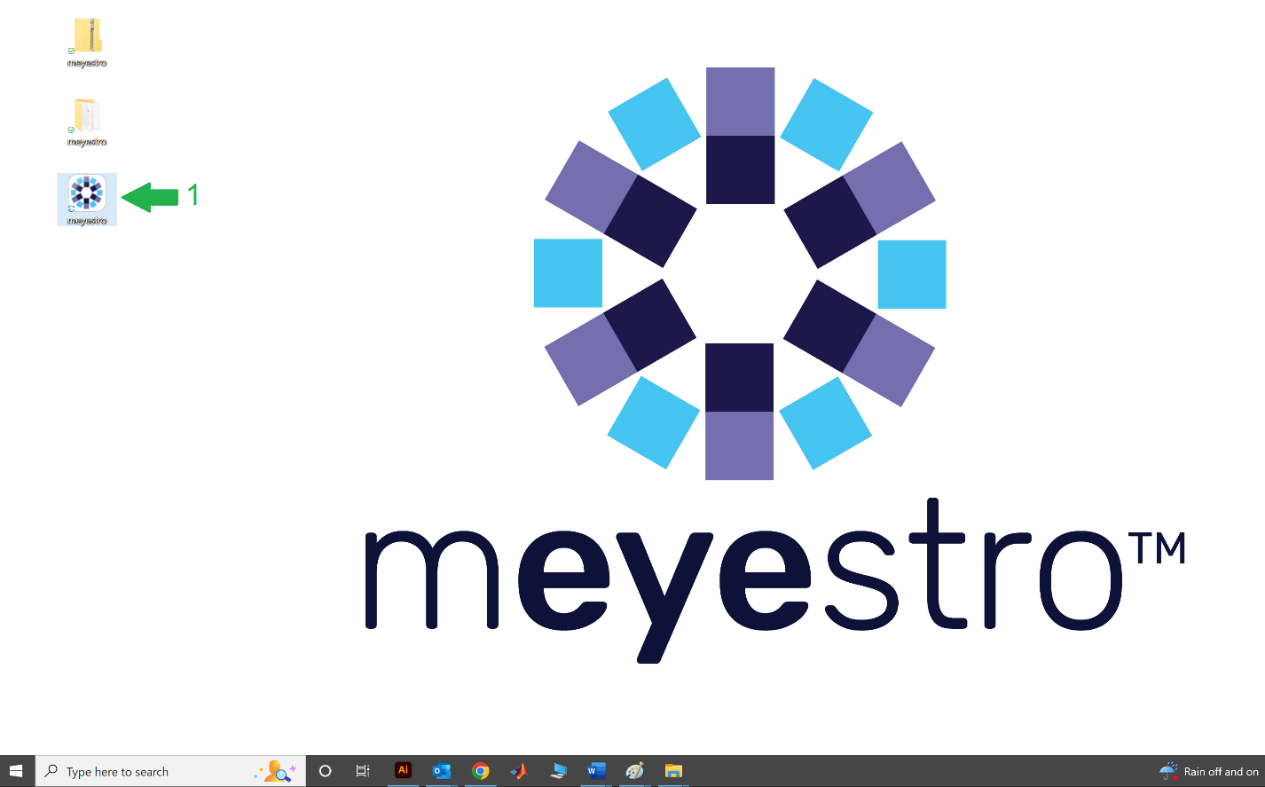


Read the warning message, and click on “Ok” (1):


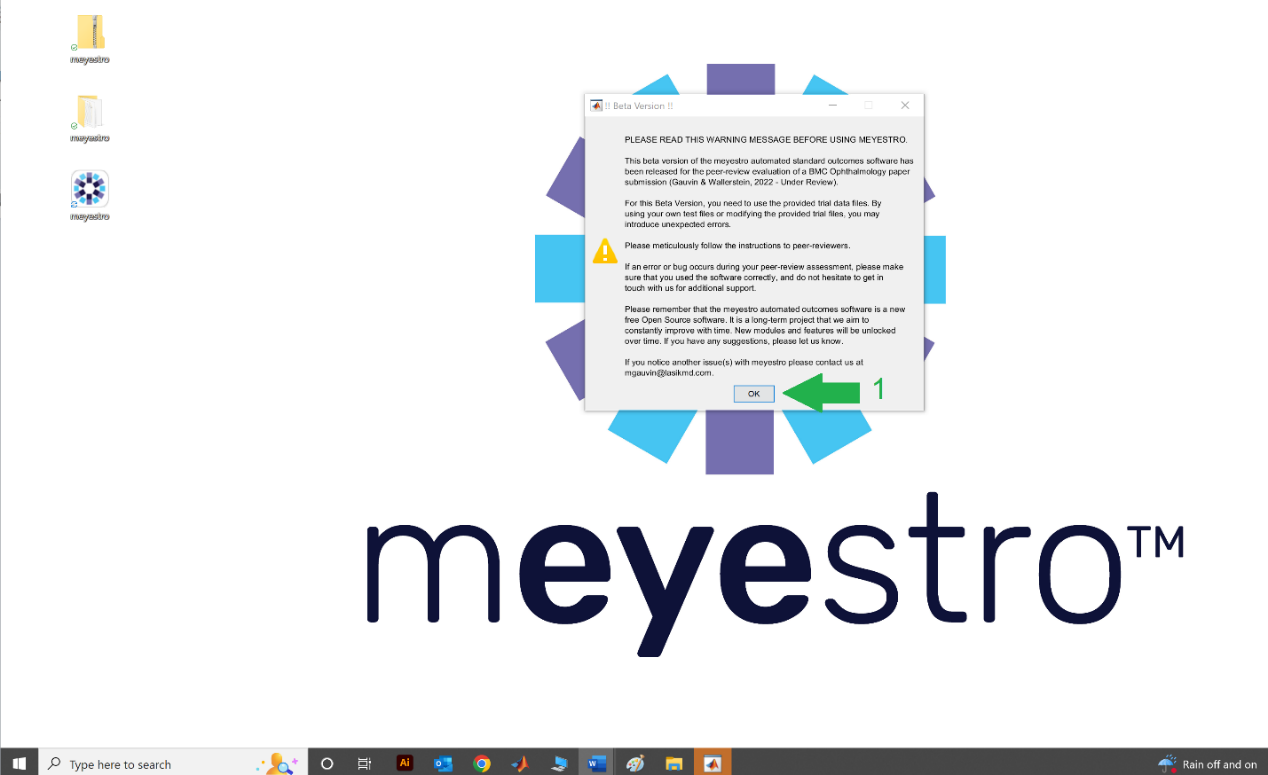


The meyestro main screen opens, and the software is ready to use:


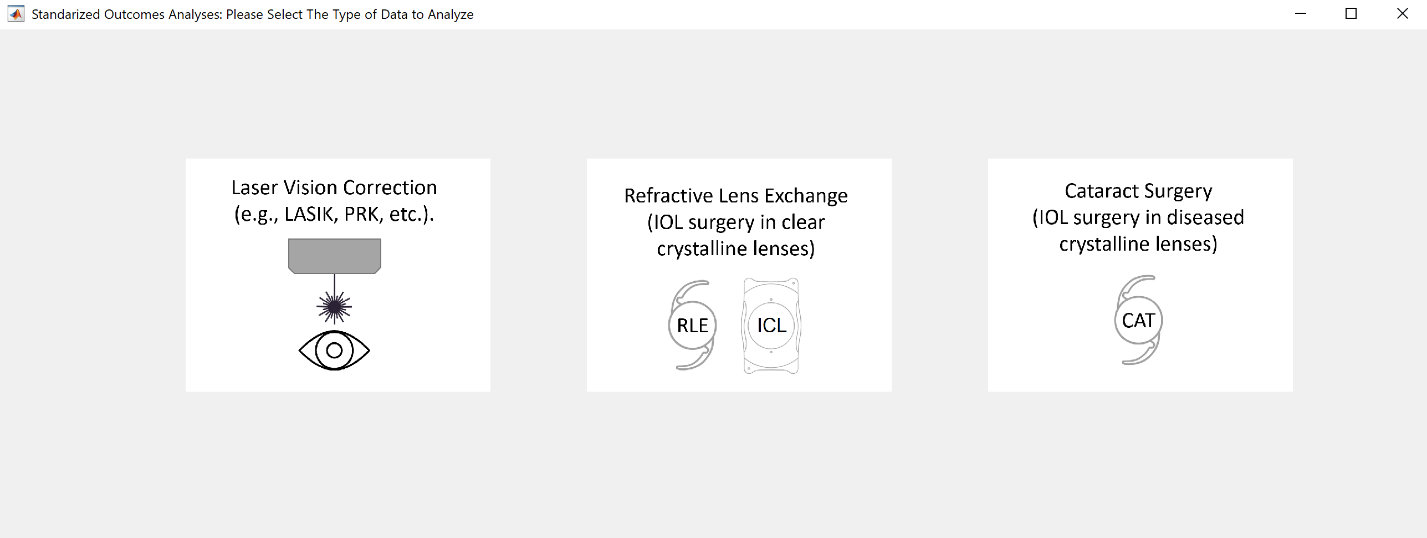


# **Demonstration using the 3 provided Trial Datasets:**

## **Trial 1**

Trial 1 includes two groups (Group A and Group B), and it is a contralateral eye study example in hyperopes. Therefore, the subjects in Group A and Group B are the same.

Start the meyestro application, and once on the main screen, click on “Laser Vision Correction” button (1):


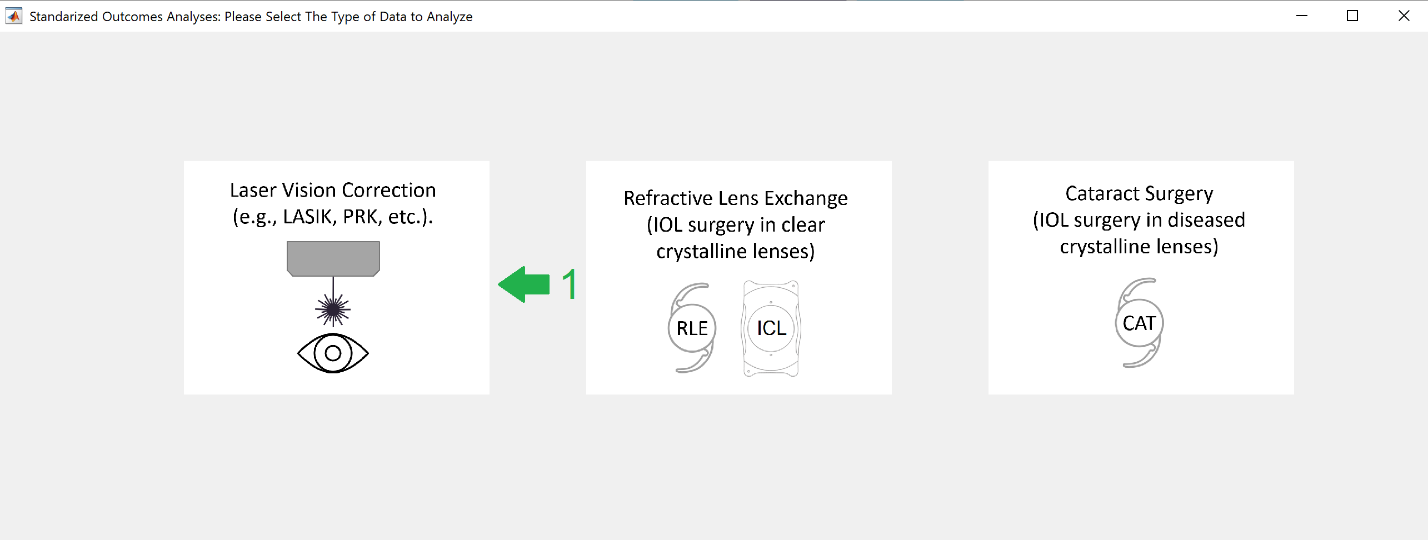


Then, click on “Two paired groups” button (1):


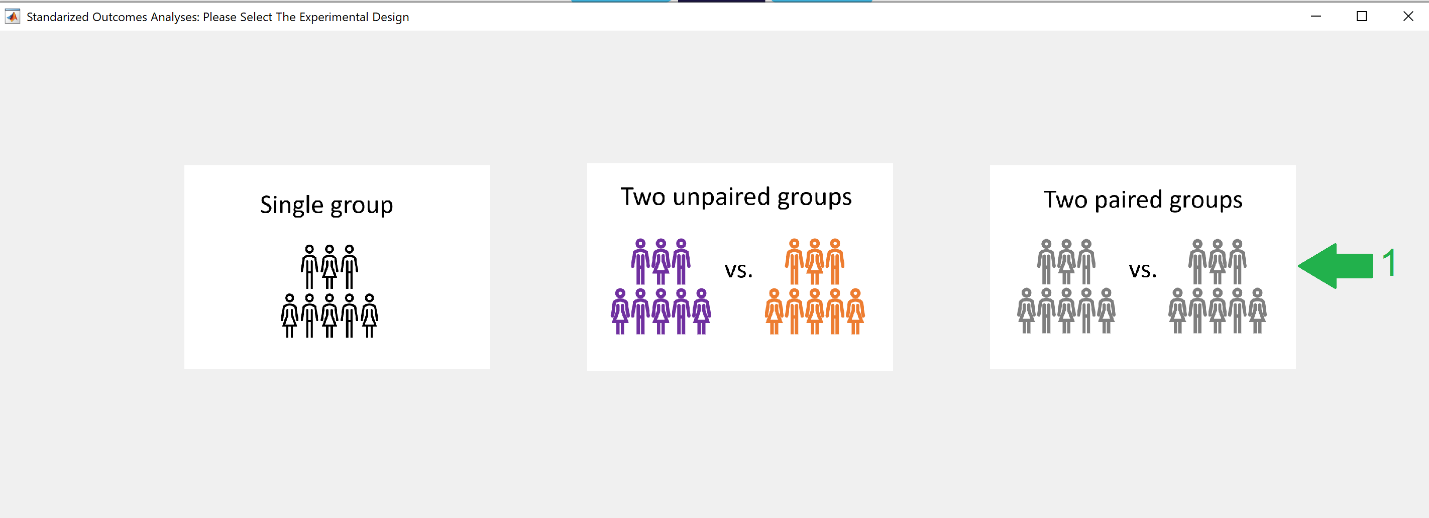


By default, meyestro will use “Group A” as the 1^st^ group name, click on “Ok” button (1):


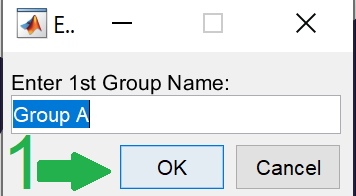


By default, meyestro will use “Group B” as the 2^nd^ group name, click on “Ok” button (1):


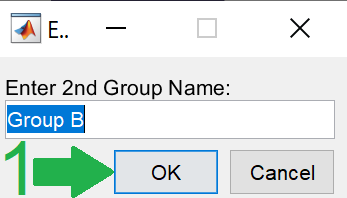


By default, meyestro will use purple as the color of the Figures for the 1st group, click on “Ok” button (1):


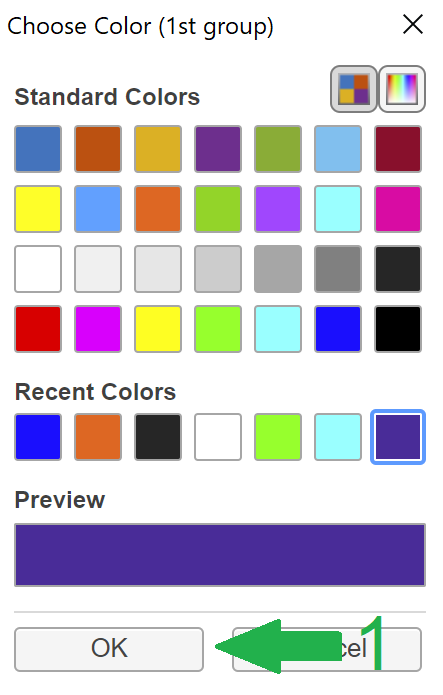


By default, meyestro will use orange as the color of the Figures for the 2^nd^ group, click on “Ok” button (1):


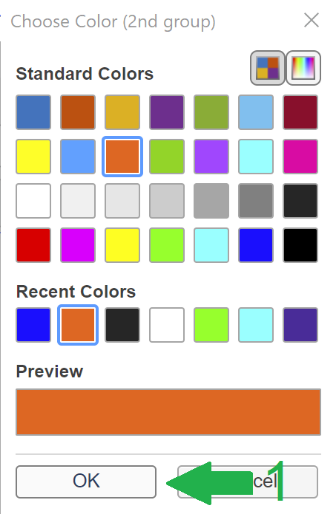


meyestro will then ask you which standard Snellen acuities to display in the cumulative postop UDVA vs. preop CDVA graph (Panel A in Figures 2, 3, and 4 in our published BMC article), select the default “20/20 20/25 20/32 20/40” option (1) and click on the “Ok” button (2):


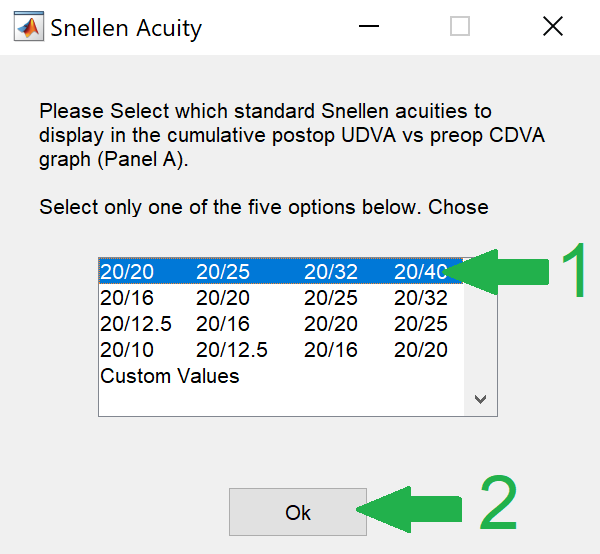


meyestro will then ask you the LogMAR threshold to be considered as being “20/20 20/25 20/32 20/40”, use the default values (0, 0.1, 0.2, and 0.3 LogMAR) and click on “Ok” button:


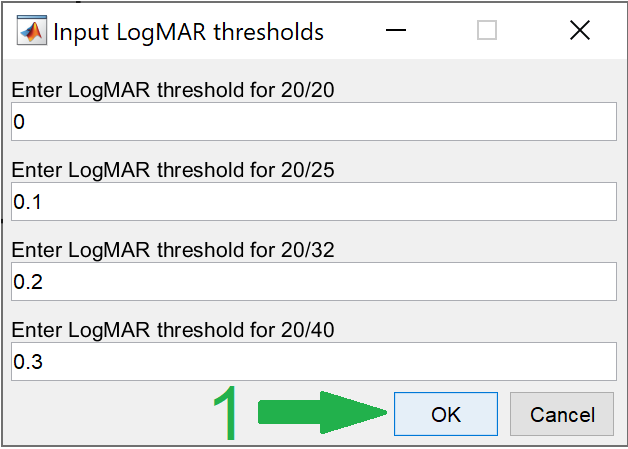
*

* This input screen is simply to tell the software what LogMAR values should be considered as 20/20, 20/25, etc. For example, a vision of 20/20 is equal to 0 LogMAR, and a vision of 20/20-2 is equal to 0.04 logMAR. If you want 20/20-2 to still be considered 20/20 in the cumulative postop UDVA vs. preop CDVA graph (Panel A in Figures 2, 3, and 4), you will need to enter 0.04 as the threshold for 20/20. For this demonstration, use the default values.

meyestro will then ask you the efficacy and safety indices Snellen level. Select the default 20/20 (1) option and click on the “Ok” button (2):


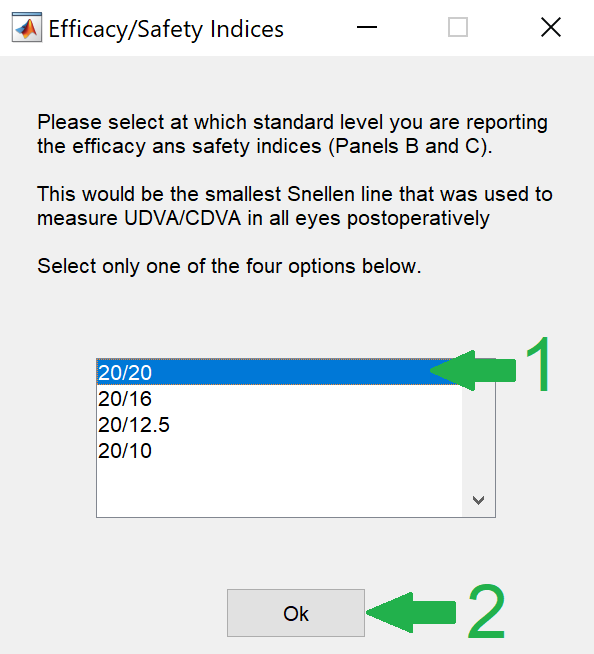
**

** This input screen is to tell the software at what standard Snellen level to calculate the efficacy and safety indices. This would be the smallest Snellen line that was used to measure UDVA/CDVA in all eyes preoperatively and postoperatively. Usually, this is 20/20 or 20/16. For example, if vision was measured down to 20/16 in all eyes preoperatively but only down to 20/20 postoperatively, then you need to use the 20/20 level because otherwise, if you select the 20/16 level, this will create the impression on panels B and C that many eyes have one line worse of UDVA than preop CDVA (low efficacy index) and lost line of CDVA (low safety index) when in fact no eyes have lines worse of UDVA or lost CDVA line, but simply vision was not measured equally preop and postop. Please select the smallest Snellen optotype you trust was used in all cases pre and postop.

meyestro will then ask if you want to include a standard stability graph. For Trial 1 datasets, the optional stability data is available, so click on “Yes” button (1):


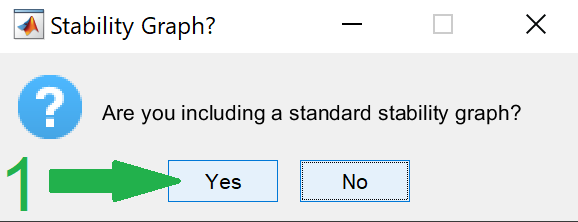


meyestro will then ask you how many distinct postoperative time points you want to include in the standard stability graph. For Trial 1 datasets, we have 5 distinct time points, (1) please select “5”, and click on the “Ok” button (2):


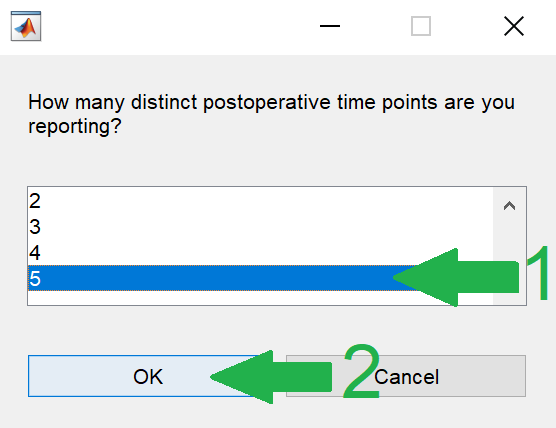


meyestro will then ask you the text label to display for each of the 5 time points. Use the default values of 1, 3, 6, 12 and 24 months, and click on the “Ok” button (1):


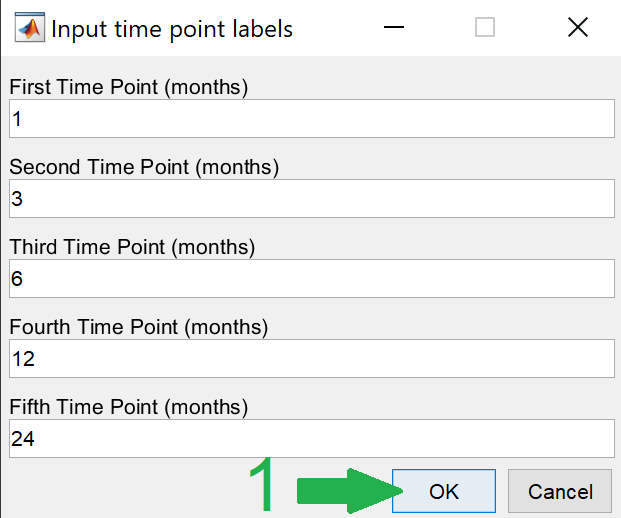


meyestro will then ask you which two time points to compare. For this Trial 1 example, (1) hold “Ctrl” and select “3” and “24”, and click on the “Ok” button (2):


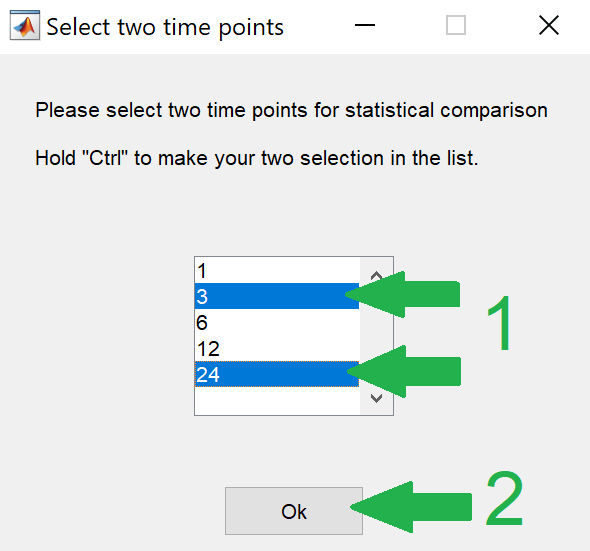


meyestro will now ask you to select the Excel data file for the 1^st^ group. (1) Navigate to the Trial 1 datasets folder, select the Group A file and click on “Open” button (2):


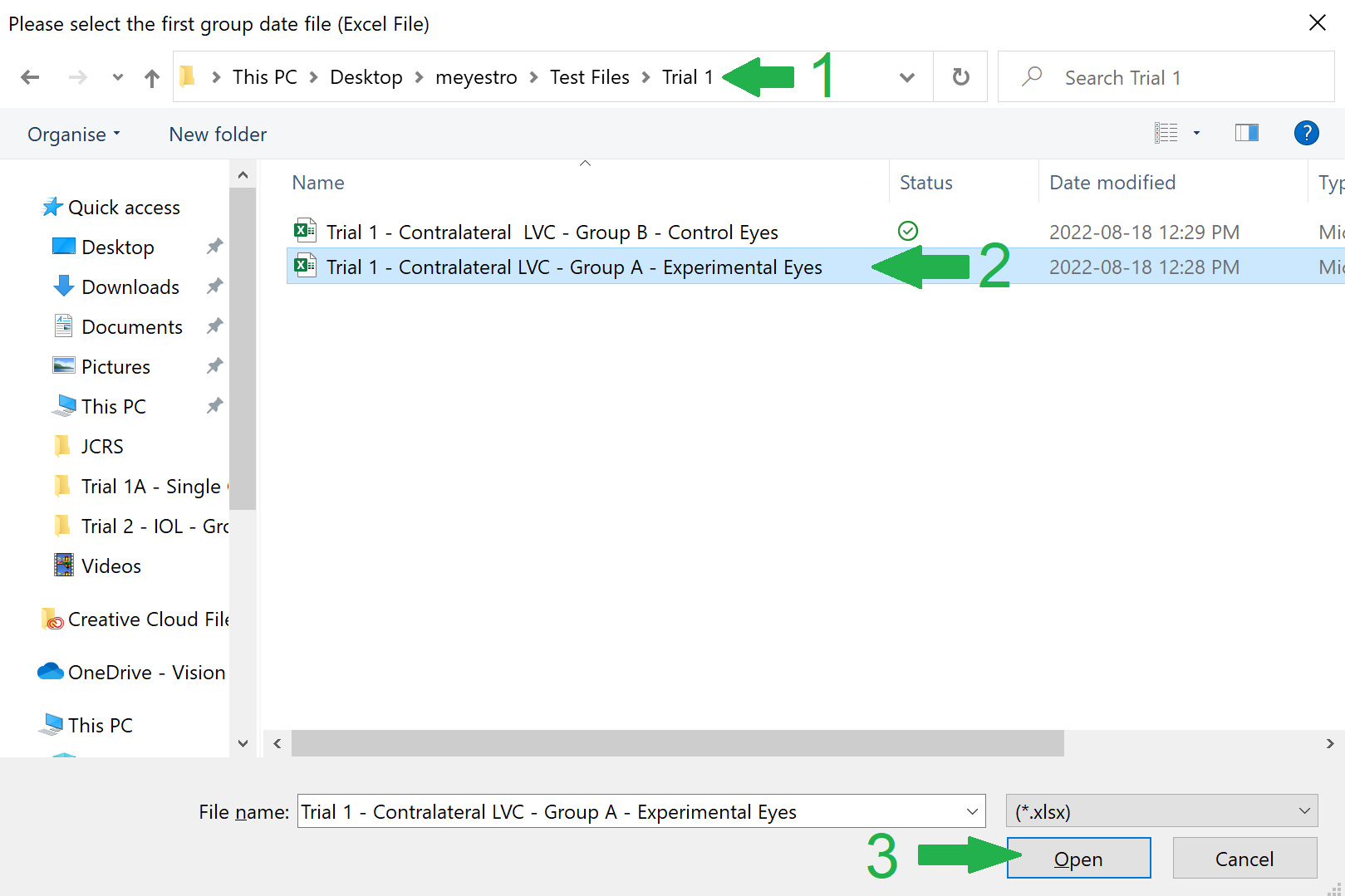


Finally, meyestro will now ask you to select the Excel data file for the 2^nd^group. (1) Navigate to the Trial 1 datasets folder, select the Group B file and click on “Open” button (2):


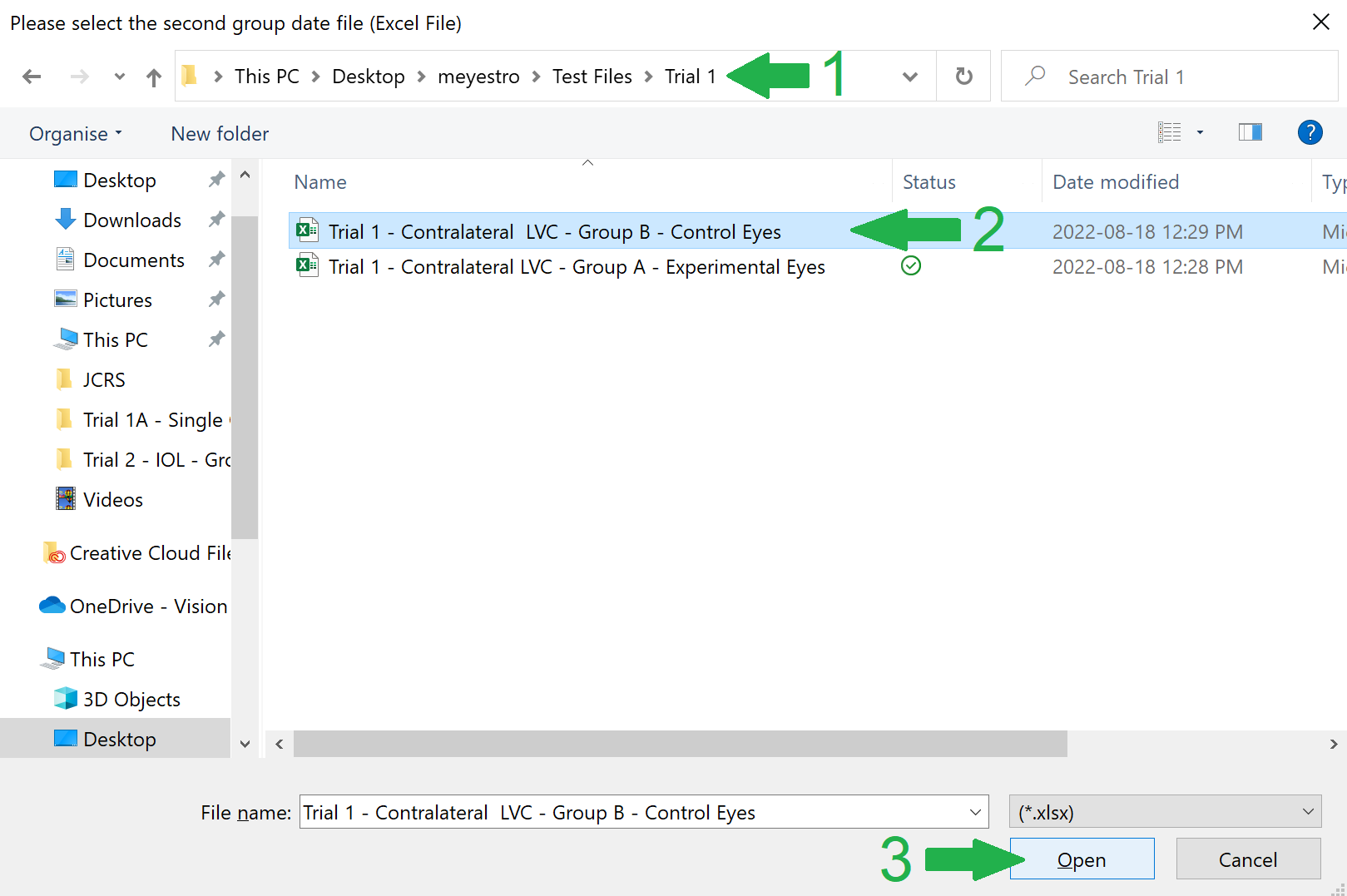


meyestro will then generate all the standard graphs and automatically save all figures as TIFF images in a folder that will automatically open. Once the images folder opens, please double-click on the AllStandardGraphs.TIFF file to open the one-page view with all graphs (the 10-figures sheet):


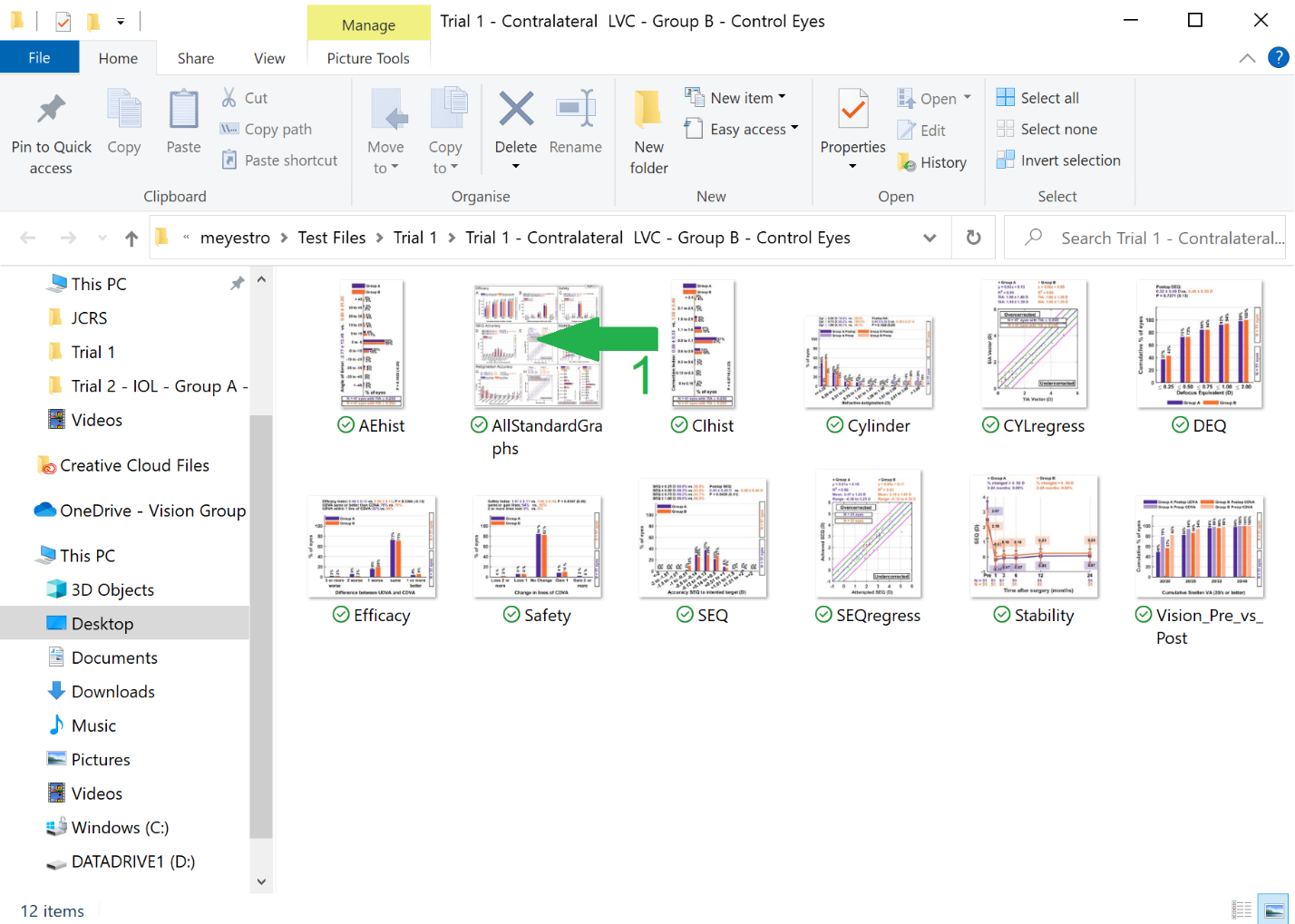


You now successfully generated all standard Figures for Trial 1. The resulting image can be included in your submission to any of the refractive surgery journals:


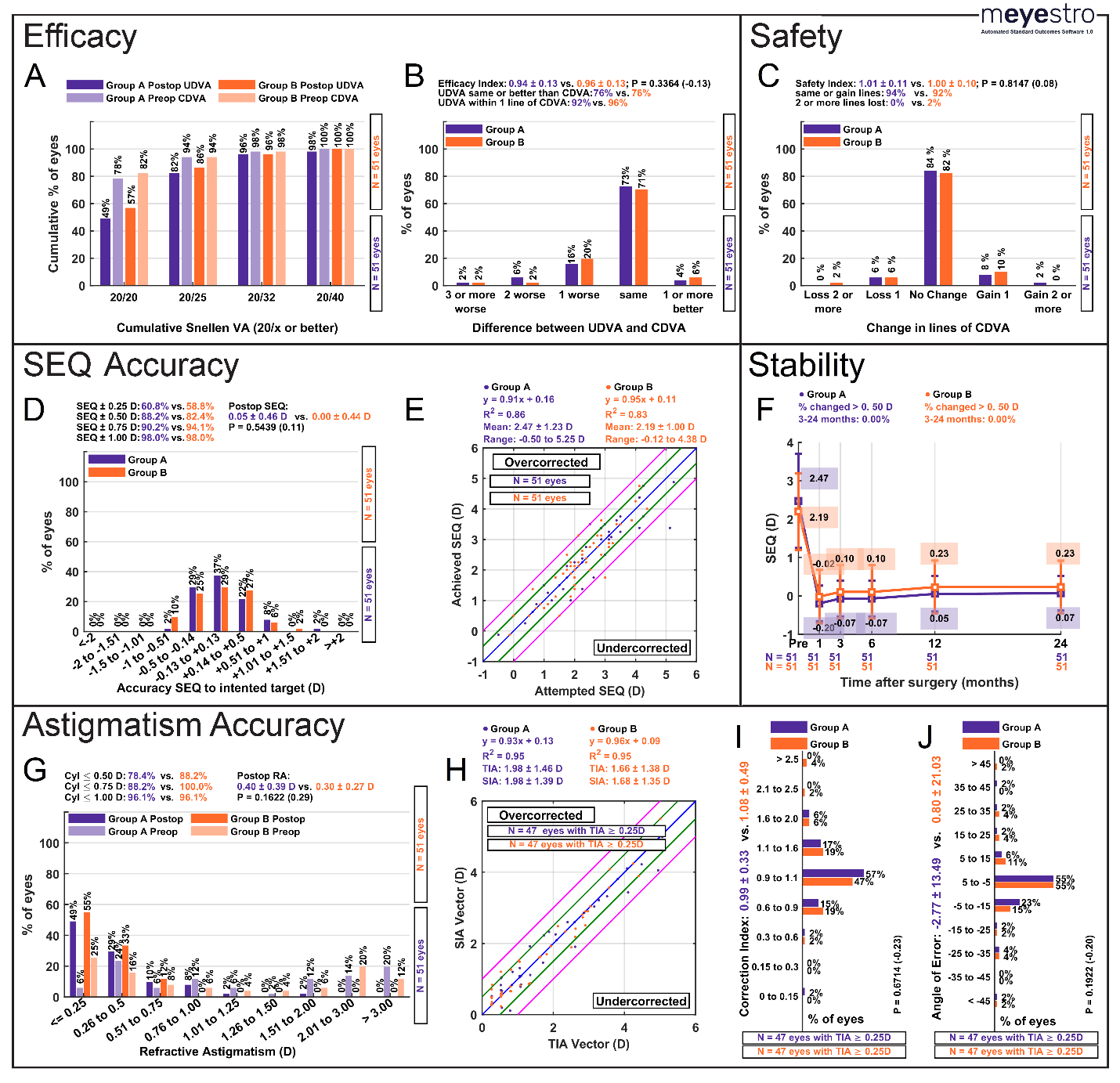


## **Trial 2**

Trial 2 includes a single group (Group A). It is a contralateral eye study example in hyperopes.

To start trial 2, open meyestro and click on “Refractive Lens Exchange” button (1):


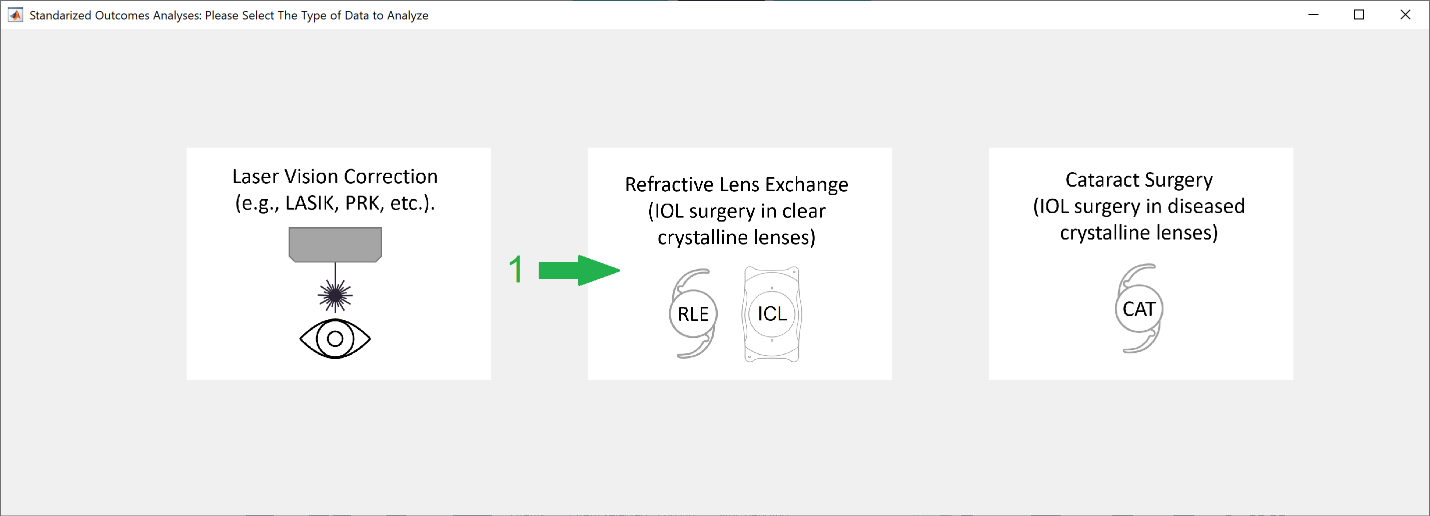


Click on “Single group” button (1):


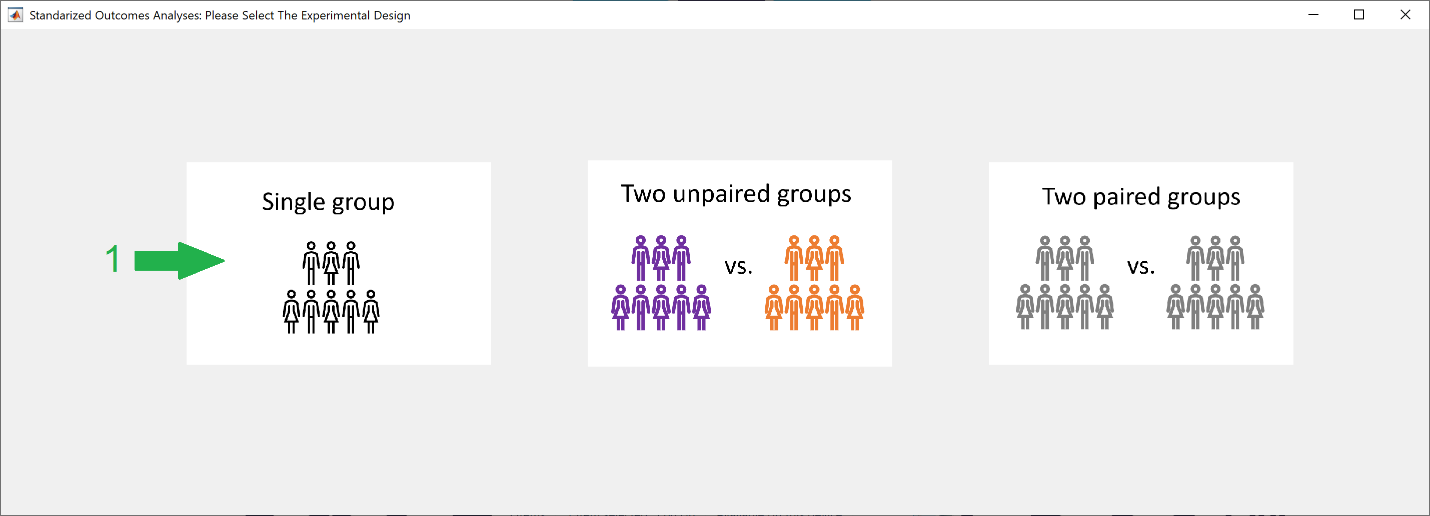


By default, meyestro will use “Group A” as the 1^st^ group name, click on “Ok” button (1)


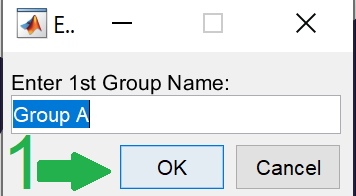


Now choose the color of the graphs. Click on the blue colored square (1) and then click on “Ok” button (2):


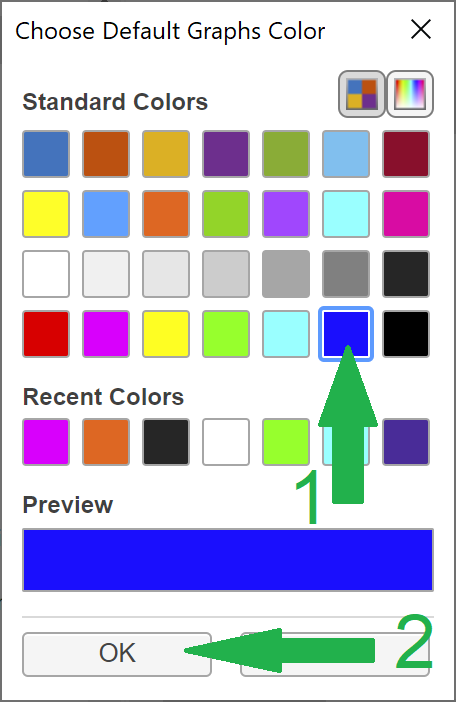


meyestro will then ask you which standard Snellen acuities to display in the cumulative postop UDVA vs. preop CDVA graph (Panel A in Figures 2, 3, and 4), select the “20/16 20/20 20/25 20/32” option (1) and click on the “Ok” button


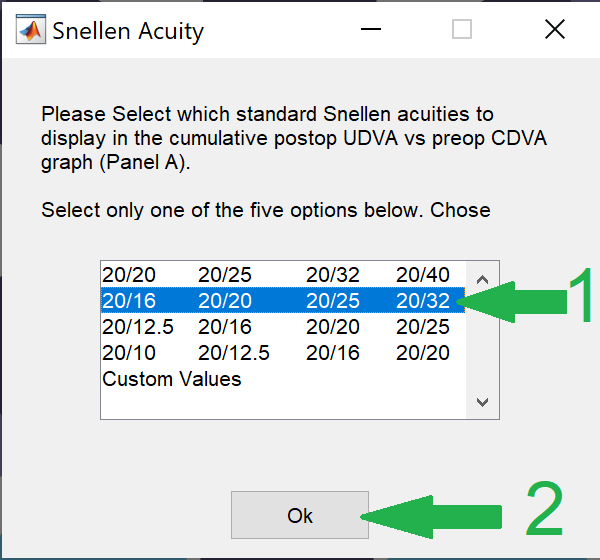


meyestro will then ask you the LogMAR threshold to be considered as being “20/16 20/20 20/25 20/32”, use the default values (-0.1, 0, 0.1, and 0.2 LogMAR) and click on “Ok” button (1):


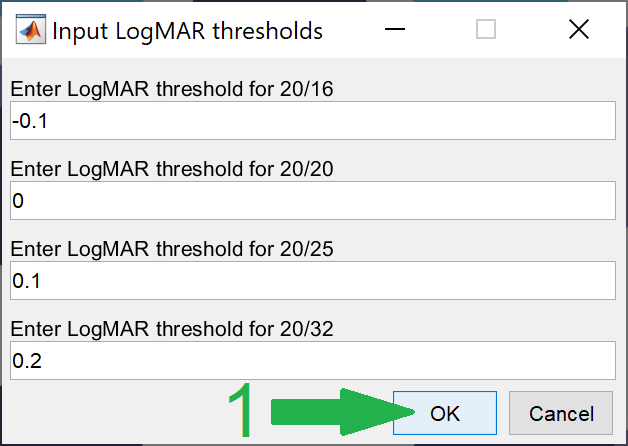
*

* This input screen is simply to tell the software what should be considered 20/16 20/20, 20/25, etc. For example, a vision of 20/16 equals -0.1 LogMAR, and a vision of 20/16-2 equals -0.06 logMAR. If you want 20/16-2 to still be considered 20/16 in the cumulative postop UDVA vs. preop CDVA graph (Panel A in Figures 2, 3, and 4), you will need to enter -0.06 as the threshold for 20/16.

meyestro will then ask you the efficacy and safety indices Snellen level. For this trial 2 example, (1) please select 20/16 and click on the “Ok” button (2):


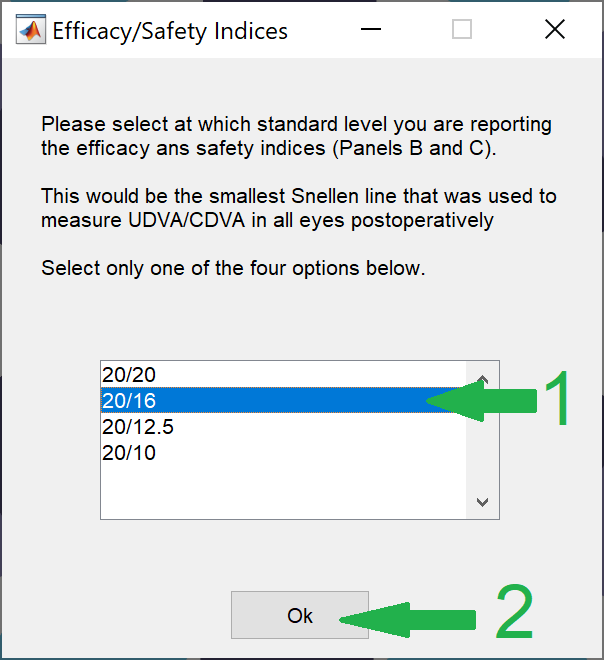
**

** This input screen is to tell the software at what standard Snellen level to calculate the efficacy and safety indices. This would be the smallest Snellen line that was used to measure UDVA/CDVA in all eyes preoperatively and postoperatively. For this Trial 2 example, select 20/16.

meyestro will then ask if you want to include a standard stability graph. For this Trial 2 example, please will click on “No” (1):


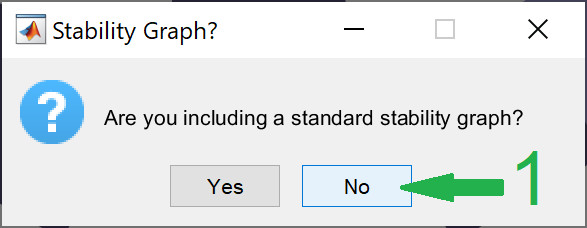


Finally, meyestro will now ask you to select the Excel data file for the group. (1) Navigate to the Trial 2 datasets folder, select the Group A file and click on “Open” button (2):


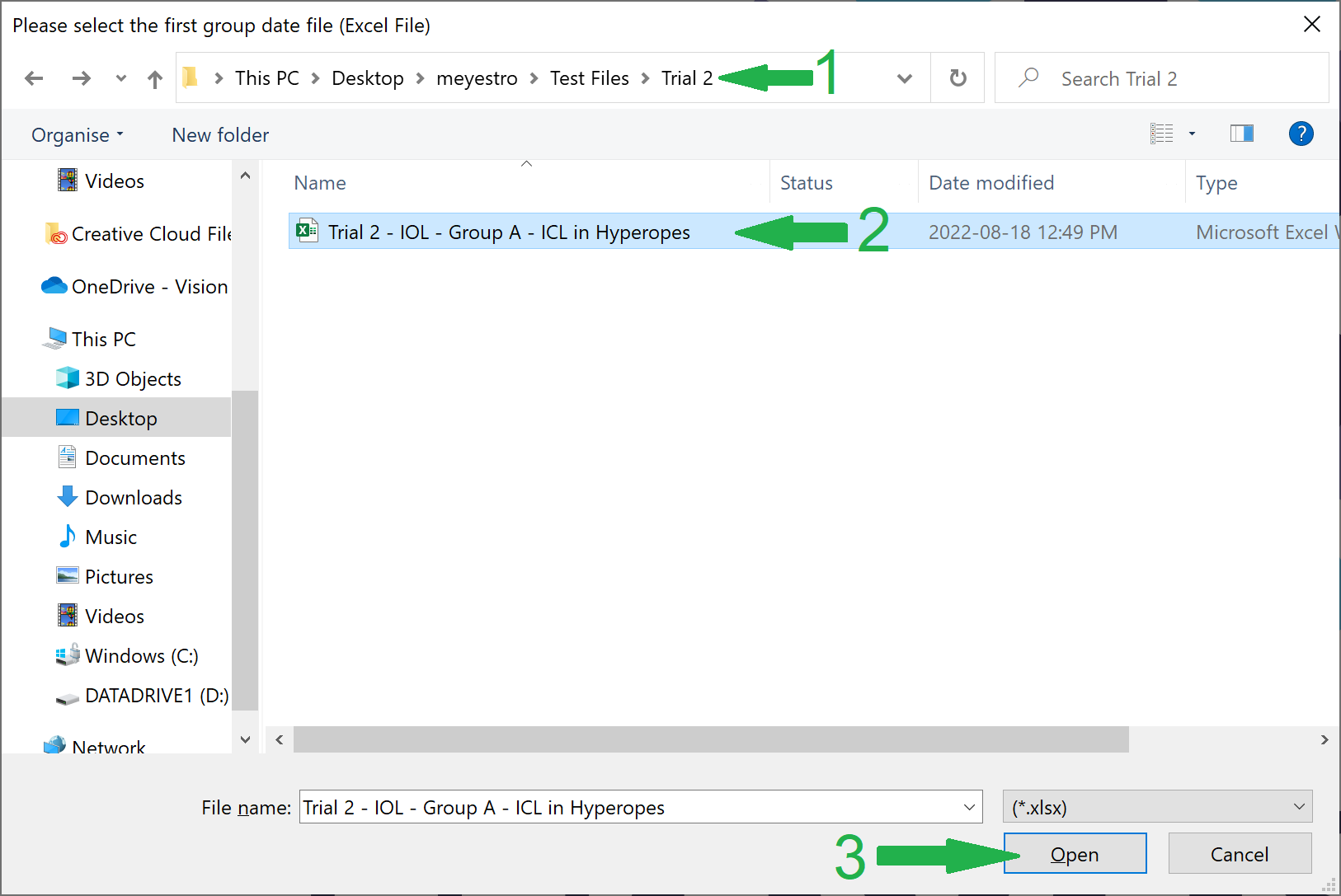


meyestro will then generate all the standard graphs and automatically save all figures as TIFF images that you can open individually. Double-click on the AllStandardGraphs.TIFF file to open the one-page view with all graphs (the 10-figures sheet):


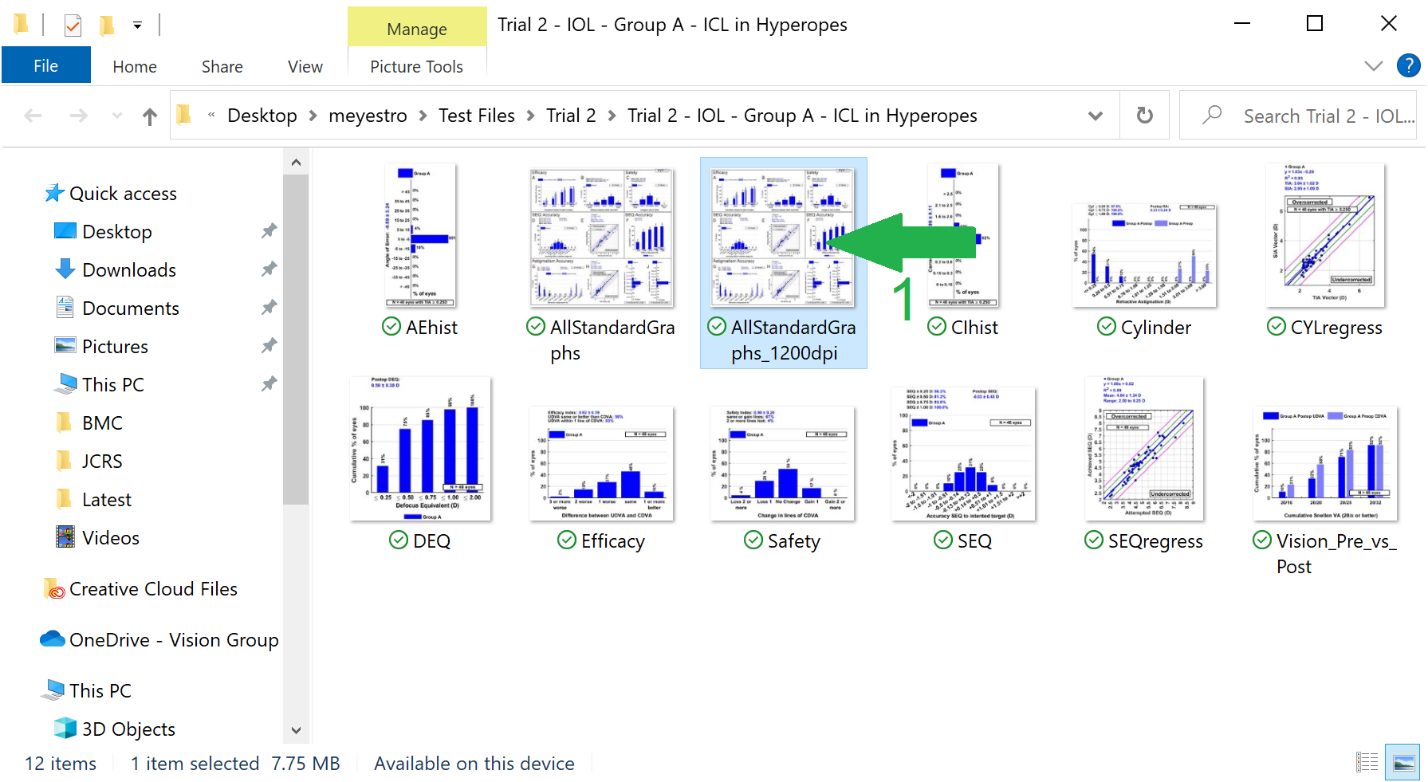


You now successfully generated all standard figures for **Trial 2**. The resulting image can be included in your submission to any of the refractive surgery journals:


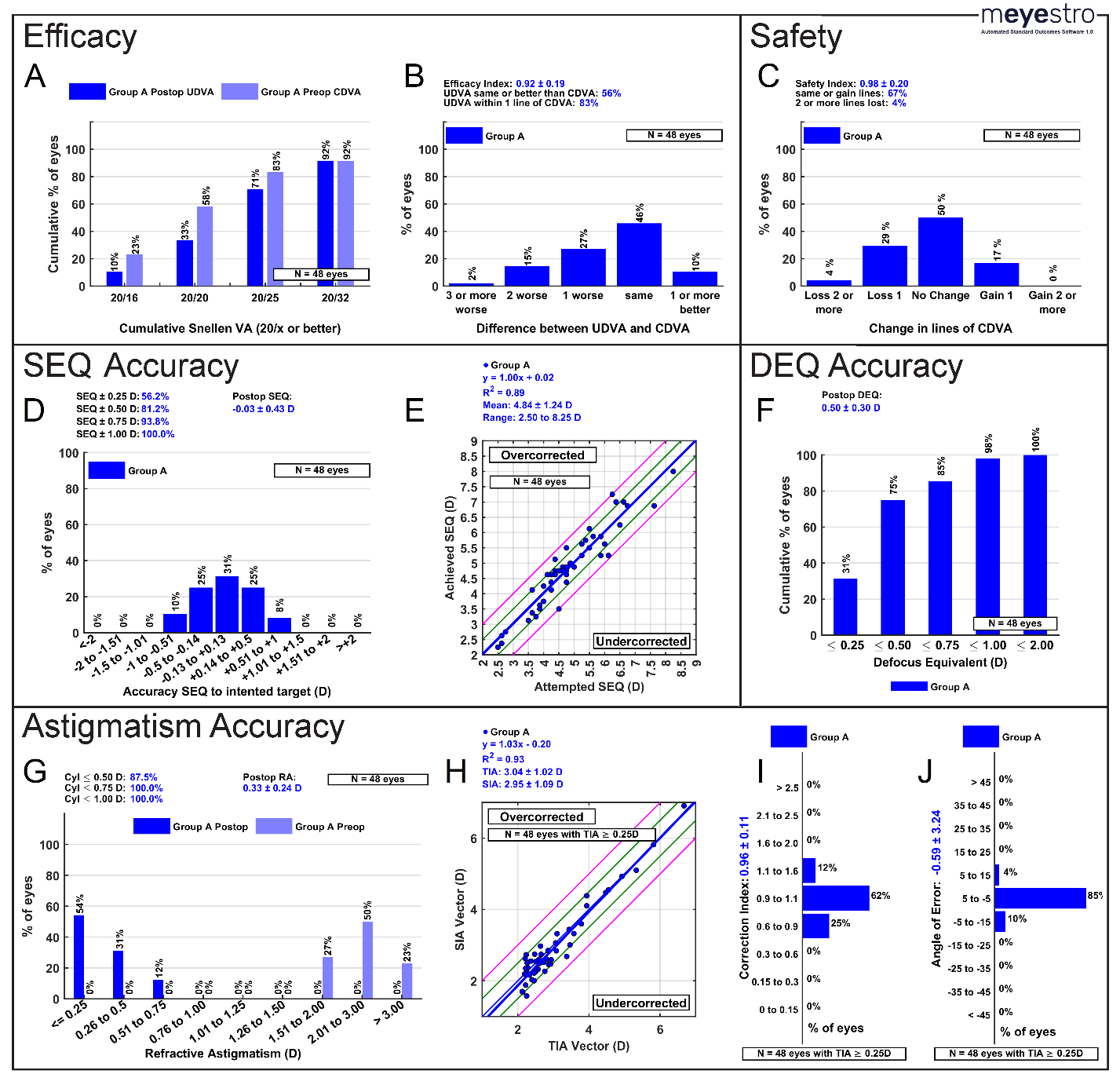


## **Trial 3**

Trial 3 includes two groups (Group A and Group B). It is a comparative myopic IOL study between two independent groups of patients with cataracts. Therefore, the subjects in Group A and Group B are not the same subjects. Those are simulated outcomes.

To start trial3, open meyestro and click on “Cataract surgery” button (1):


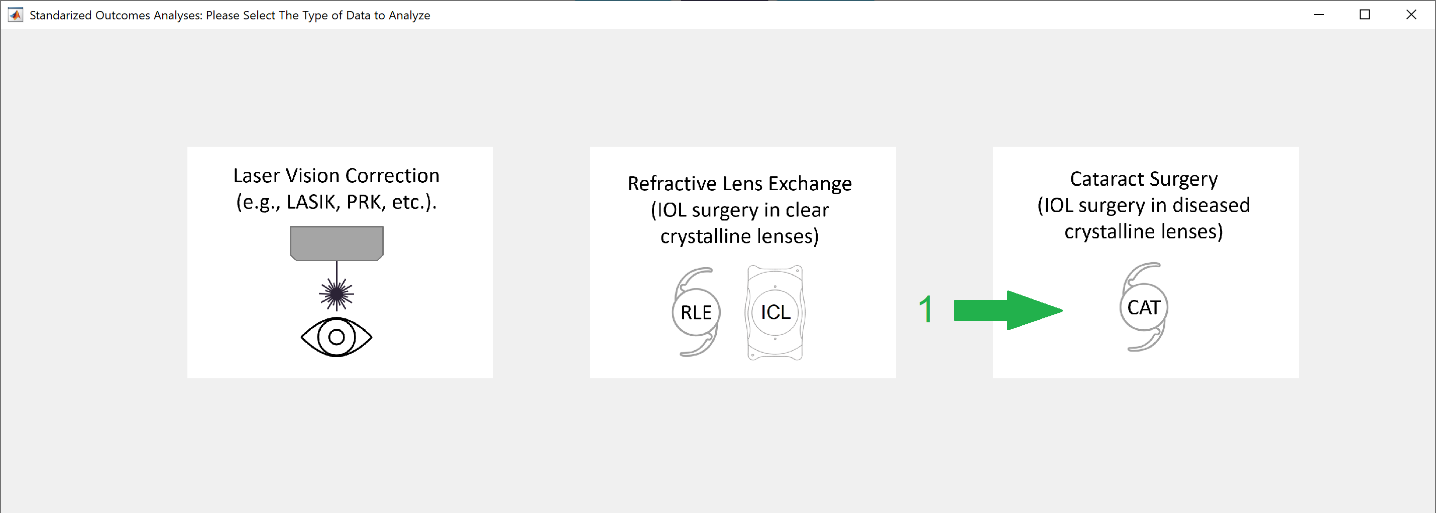


Click on “Single group” button (1):


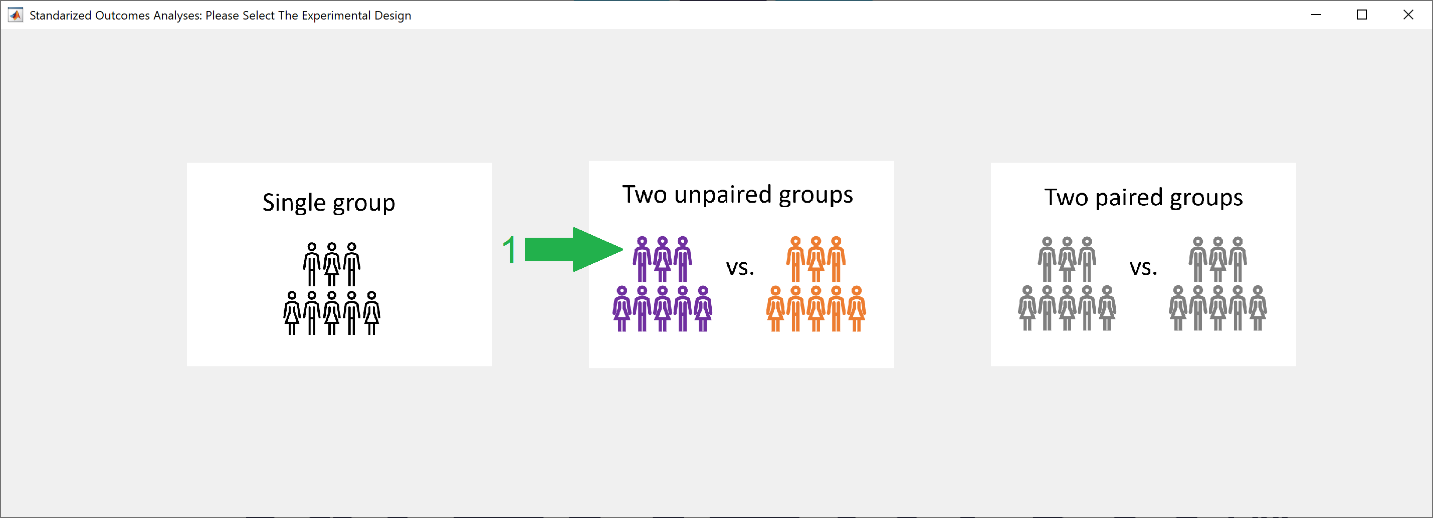


By default, meyestro will use “Group A” as the 1^st^ group name, click on “Ok” button (1):


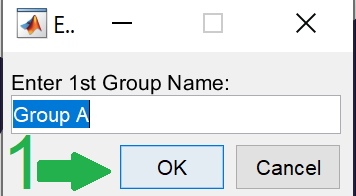


By default, meyestro will use “Group B” as the 2^nd^ group name, click on “Ok” button (1):


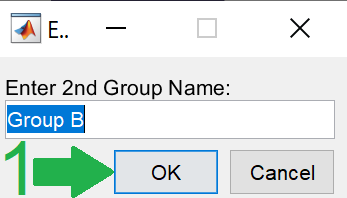


By default, meyestro will use purple as the color of the Figures for the 1st group, click on “Ok” button (1):


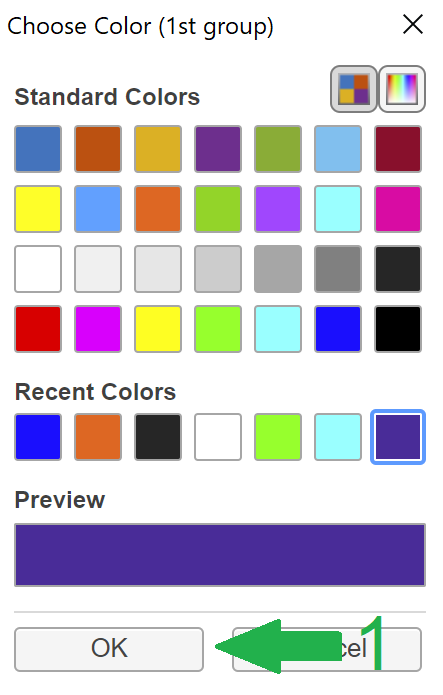


By default, meyestro will use orange as the color of the Figures for the 2^nd^ group, click on “Ok” button (1):


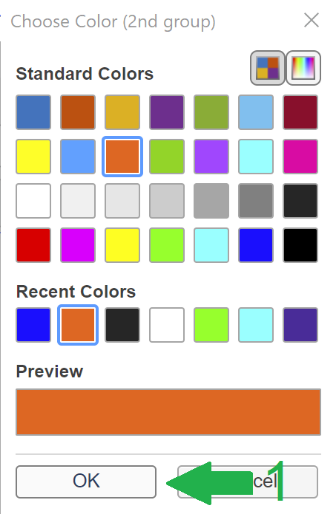


meyestro will then ask you which standard Snellen acuities to display in the cumulative postop UDVA vs. preop CDVA graph (Panel A in Figures 2, 3, and 4 in our published BMC article), select the default “20/20 20/25 20/32 20/40” option (1) and click on the “Ok” button (2):


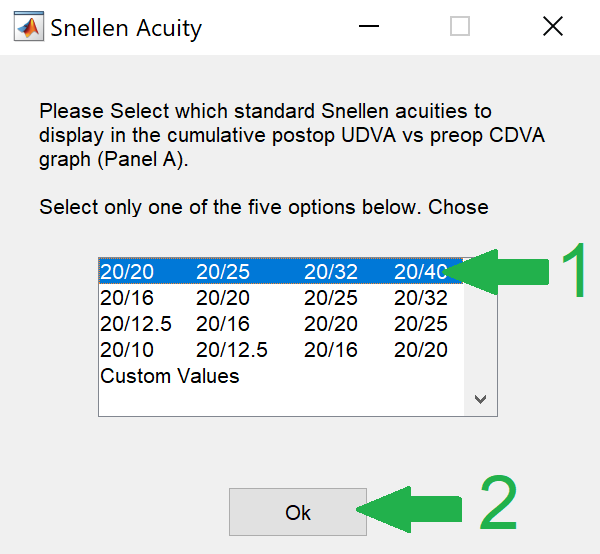


meyestro will then ask you the LogMAR threshold to be considered as being “20/20 20/25 20/32 20/40”, use the default values (0, 0.1, 0.2, and 0.3 LogMAR) and click on “Ok” button:


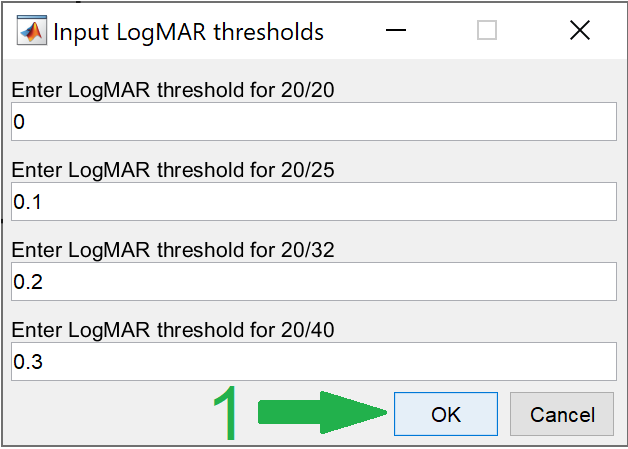
*

* This input screen is simply to tell the software what LogMAR values should be considered as 20/20, 20/25, etc. For example, a vision of 20/20 is equal to 0 LogMAR, and a vision of 20/20-2 is equal to 0.04 logMAR. If you want 20/20-2 to still be considered 20/20 in the cumulative postop UDVA vs. preop CDVA graph (Panel A in Figures 2, 3, and 4), you will need to enter 0.04 as the threshold for 20/20. For this demonstration, use the default values.

meyestro will then ask you the efficacy and safety indices Snellen level. Select the default 20/20 (1) option and click on the “Ok” button (2):


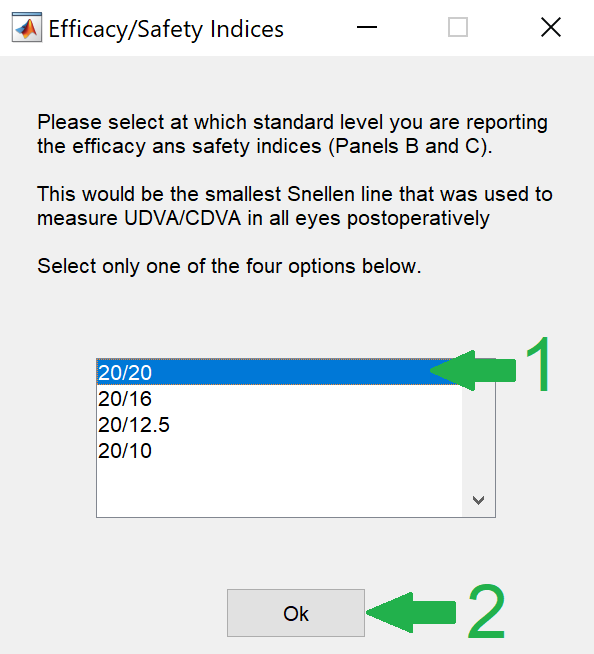
**

** This input screen is to tell the software at what standard Snellen level to calculate the efficacy and safety indices. This would be the smallest Snellen line that was used to measure UDVA/CDVA in all eyes preoperatively and postoperatively. Usually, this is 20/20 or 20/16. For example, if vision was measured down to 20/16 in all eyes preoperatively but only down to 20/20 postoperatively, then you need to use the 20/20 level because otherwise if you select the 20/16 level, this will create the impression on panel B and C that many eyes have one line worse of UDVA than preop CDVA (low efficacy index) and lost line of CDVA (low safety index) when in fact no eyes have lines worse of UDVA or lost CDVA line, but simply vision was not measured equally preop and postop. Please select the smallest Snellen optotype you trust was used in all cases pre and postop.

meyestro will then ask you if you want to include a standard stability graph. For this Trial 2 example, please will click on “No” (1):


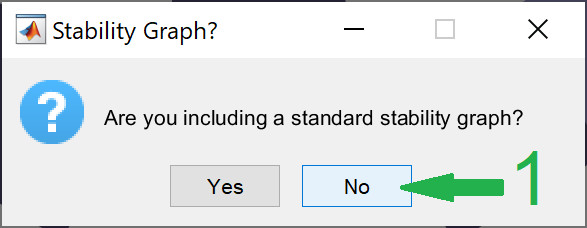


meyestro will now ask you to select the Excel data file for the group. (1) Navigate to the Trial 3 datasets folder, select the Group A file and click on “Open” button (2):


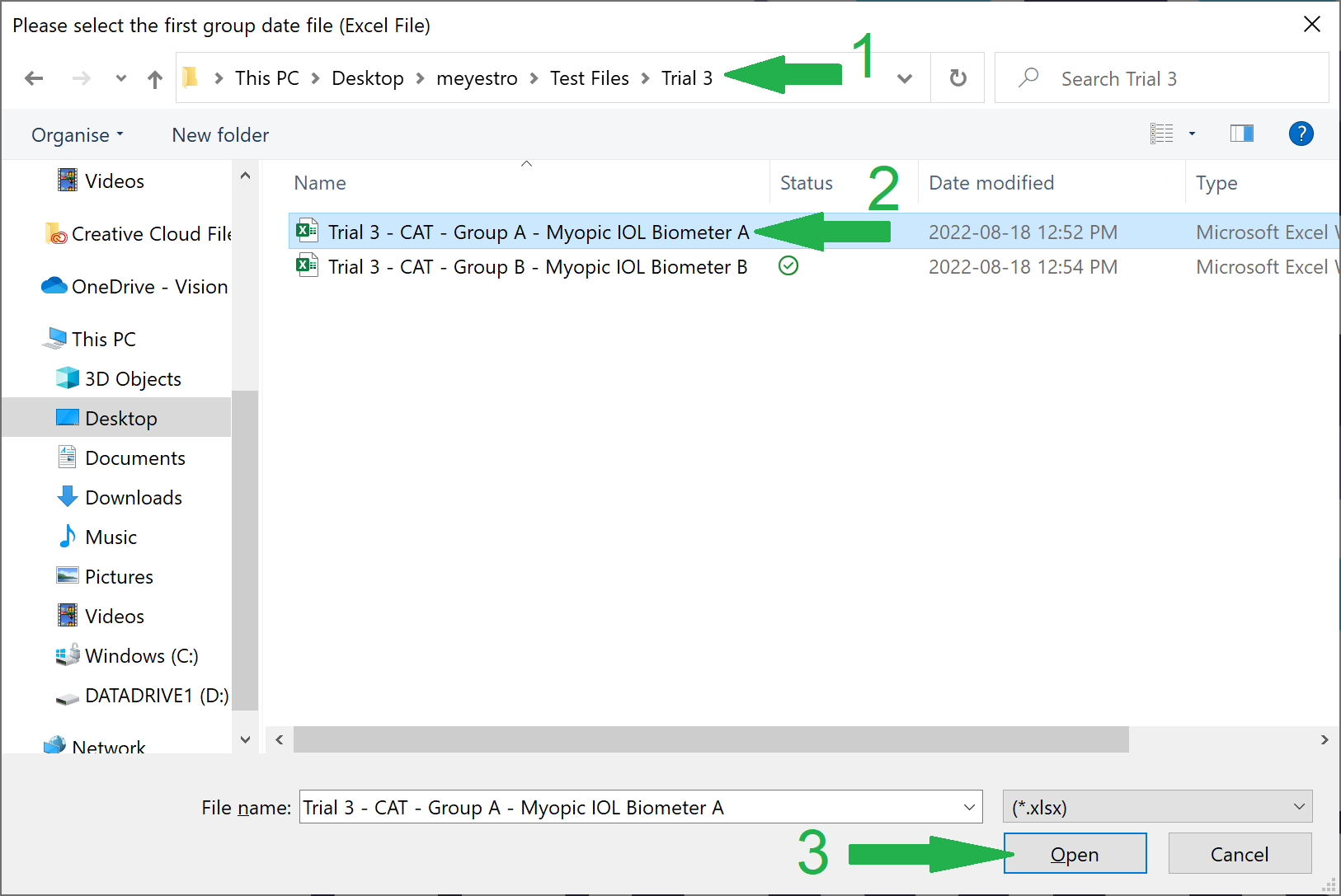


Finally, meyestro will now ask you to select the Excel data file for the 2nd group. (1) Navigate to the Trial 3 datasets folder, select the Group B file and click on “Open” button (2):


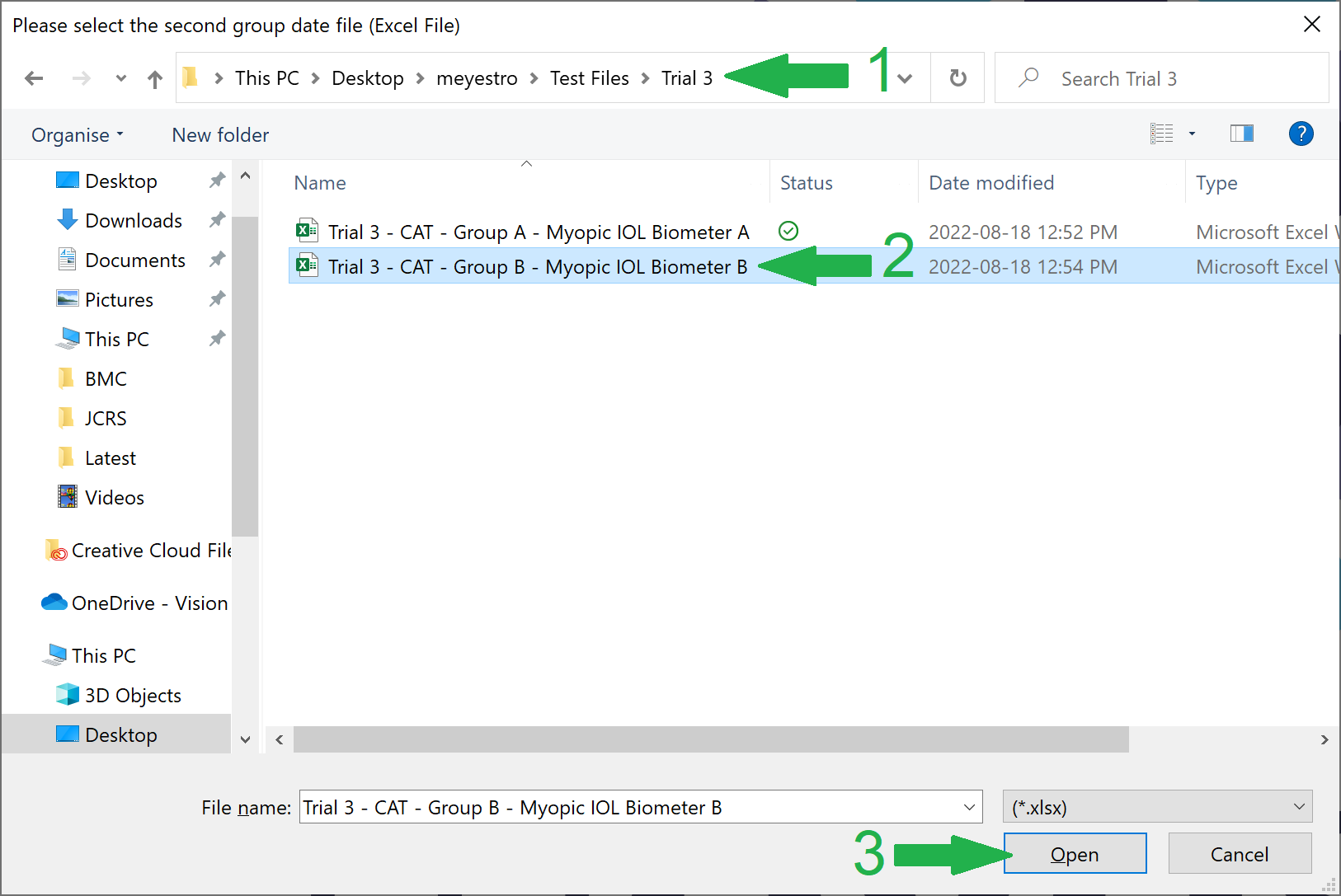


meyestro will then generate all the standard graphs and automatically save all figures as TIFF images that you can open individually. Double-click on the AllStandardGraphs_1200dpi.TIFF file to open the one-page view with all graphs with a 1200 dpi resolution (the 10-figures sheet):


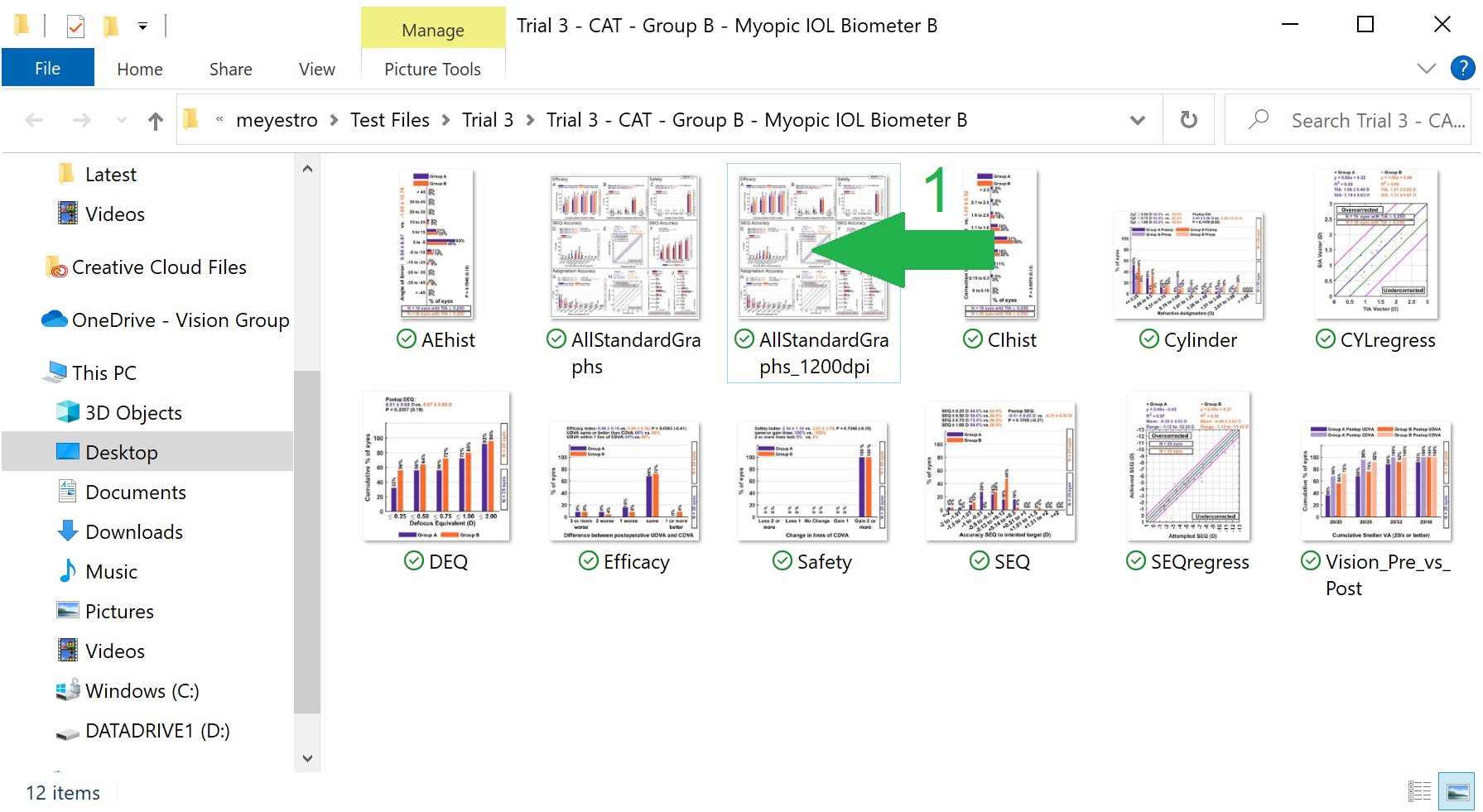


You now successfully generated all standard Figures for Trial 3. The resulting image can be included in your submission to any of the refractive surgery journals:


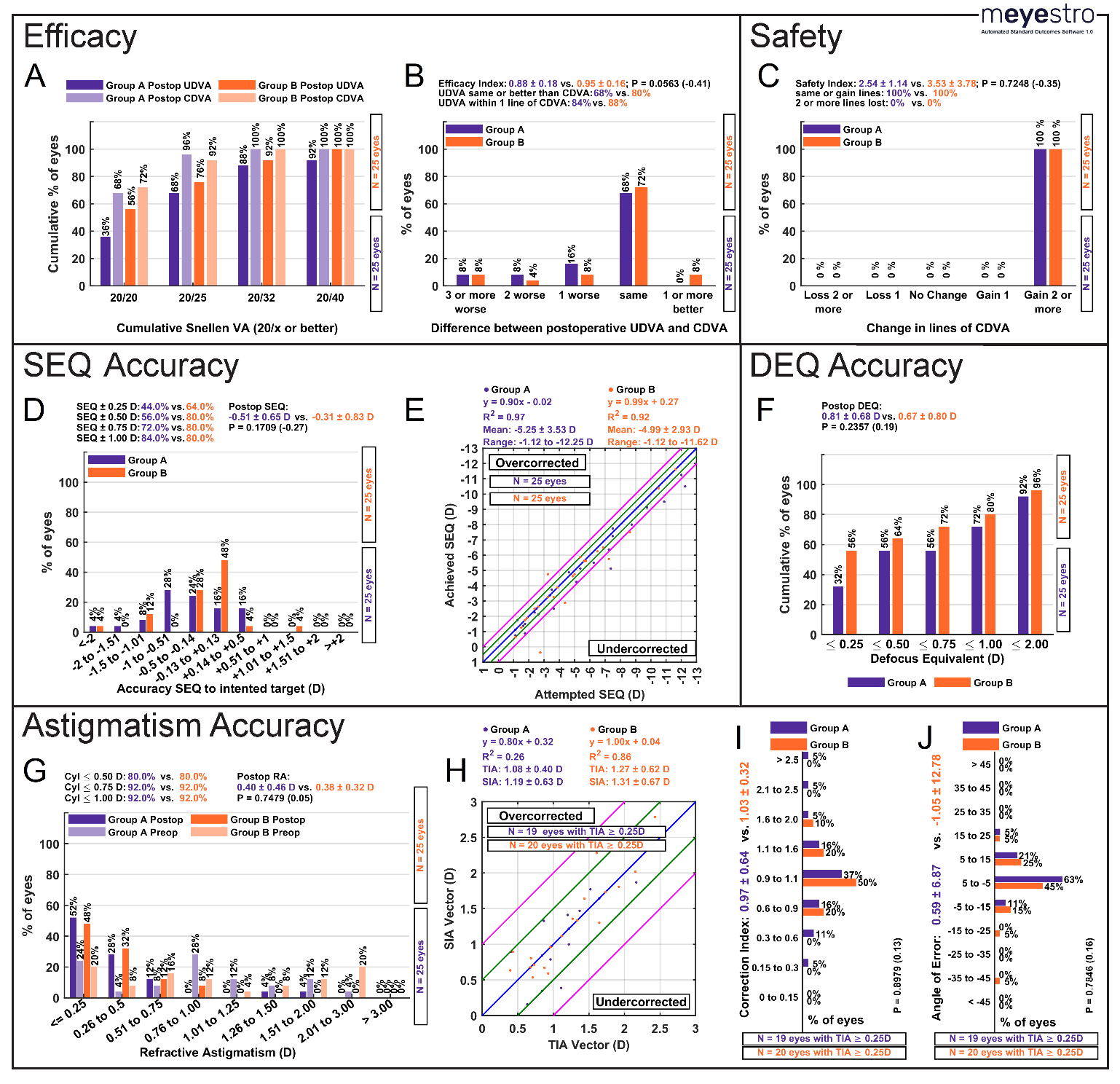


# **Optimal customizations**

Few customization options are available in meyestro.

## **Displaying custom Snellen lines in Panel A**

The user can customize which four Snellen lines to show in Panel A. Once invited to select your Snellen acuity line to display, click on “Custom Values” (1), and then click on “Ok” (2):


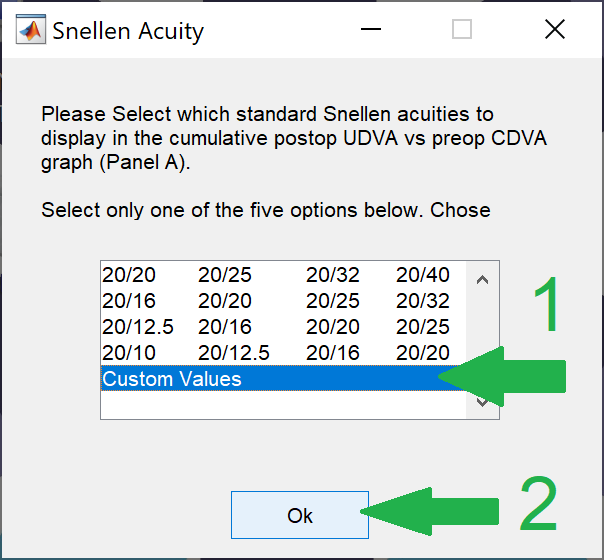


Then, enter your custom text labels to display for each Snellen line (1, 2, 3, 4), and click on “Ok” (5). In this example the user chose 20/20, 20/40, 20/80, and 20/100:


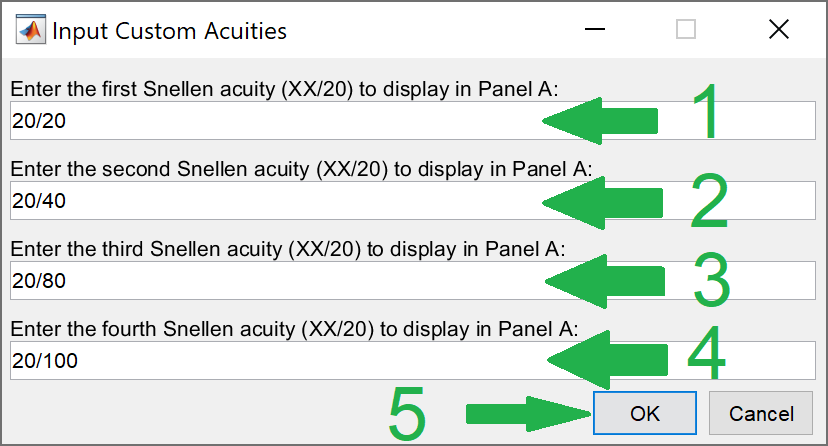


meyestro will then ask you the LogMAR threshold to be considered as being “20/20 20/40 20/80 20/100”, this would correspond to 0, 0.3, 0.6, and 0.7 LogMAR, enter those values (1, 2, 3, 4) and click on “Ok” button (5):


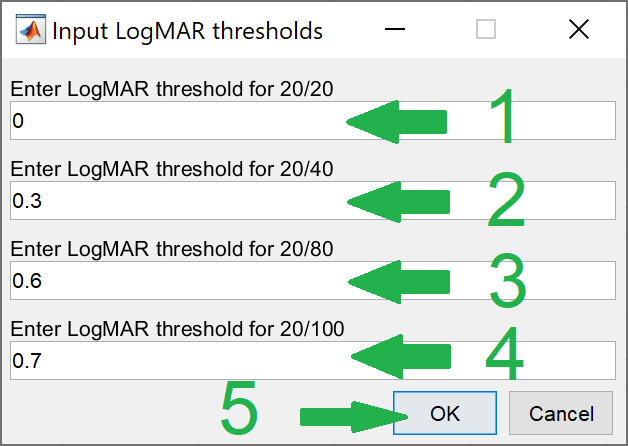


Your graph will now show your custom lines 20/20, 20/40, 20/80 and 20/100:


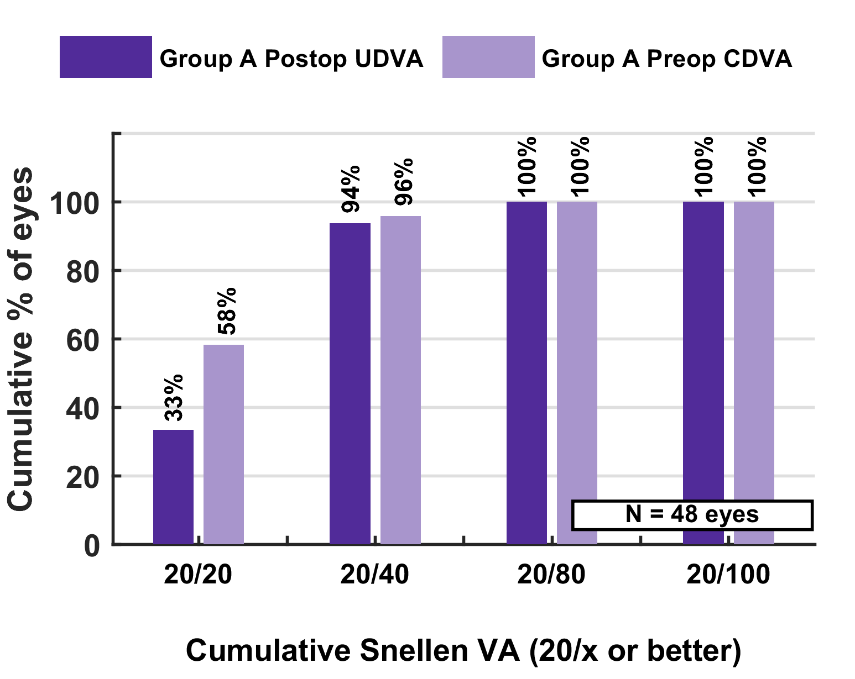


## **Customizing or creating your own figure layout**

Once you generate a set of graphs, they will be saved individually in a folder. This folder automatically opens once meyestro is done producing the figures. The user can then choose to recombine any of those graphs in their preferred image editing software. For example, the user could select for graphs highlighted in green below:


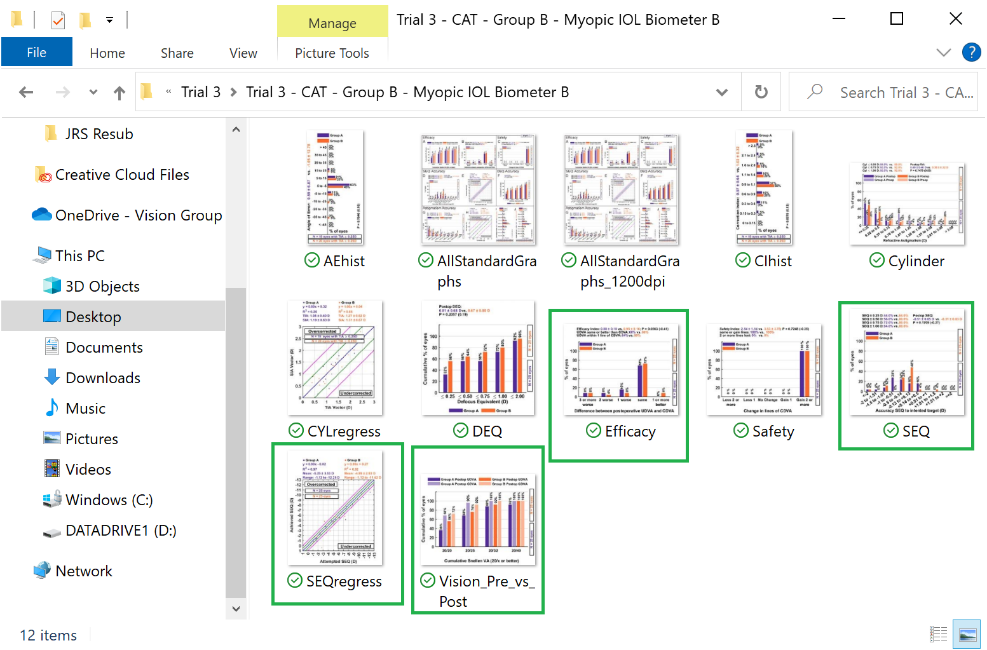


The user can now combine any of those individual graphs into their own figure layout. In this example, Microsoft Pain was used to recombine four individual figures:


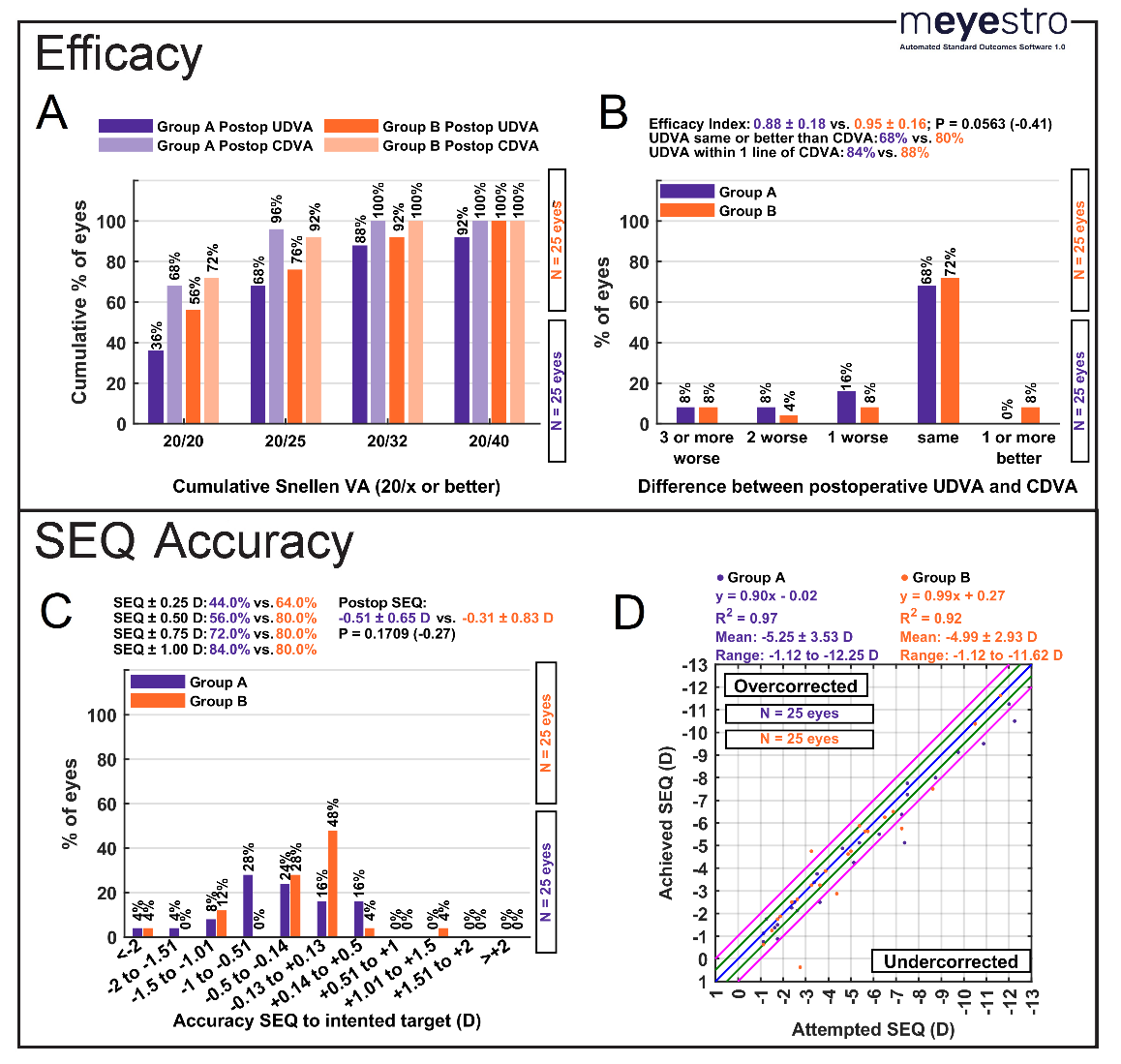


## **Future customizations**

meyestro is currently limited to the above-defined customizations. Future versions might also add additional customization. For example, meyestro currently displays up to four Snellen optotype lines of UDVA and CDVA in Panel A. In a future release it is possible that the user might be able to include more lines, for example up to six different lines instead of the current four. We might add the display in in LogMAR or in other Snellen format such as metric (6/X). We might also add a nomogram tool, or a preoperative comparison table, etc. If you have any suggestions and ideas, please contact us at [mgauvin@lasikmd.com](mailto:mgauvin@lasikmd.com).

##

## **Optional LogMAR to Snellen denominator (20/XX) conversion**

For users that use logMAR in their electronic medical record (EMR), a LogMAR to Snellen denominator automatic conversion table is included in **Supplementary File B** of our published BMC article. This automatic conversion table can be used as needed to make automatic conversion of LogMAR values to 20/XX Snellen denominator values. The converted value can simply be pasted in a mEYEstro data file.

To use the converter simply enter LogMar values in the left column (1), using decimal point (-0.1, 0, 0.3, etc). The LogMAR value will then be automatically converted and will be disaplayed in the right column in Snellen denominator (20/XX). Use those converted value (2) in your mEYEstro datafile and run your analyses.


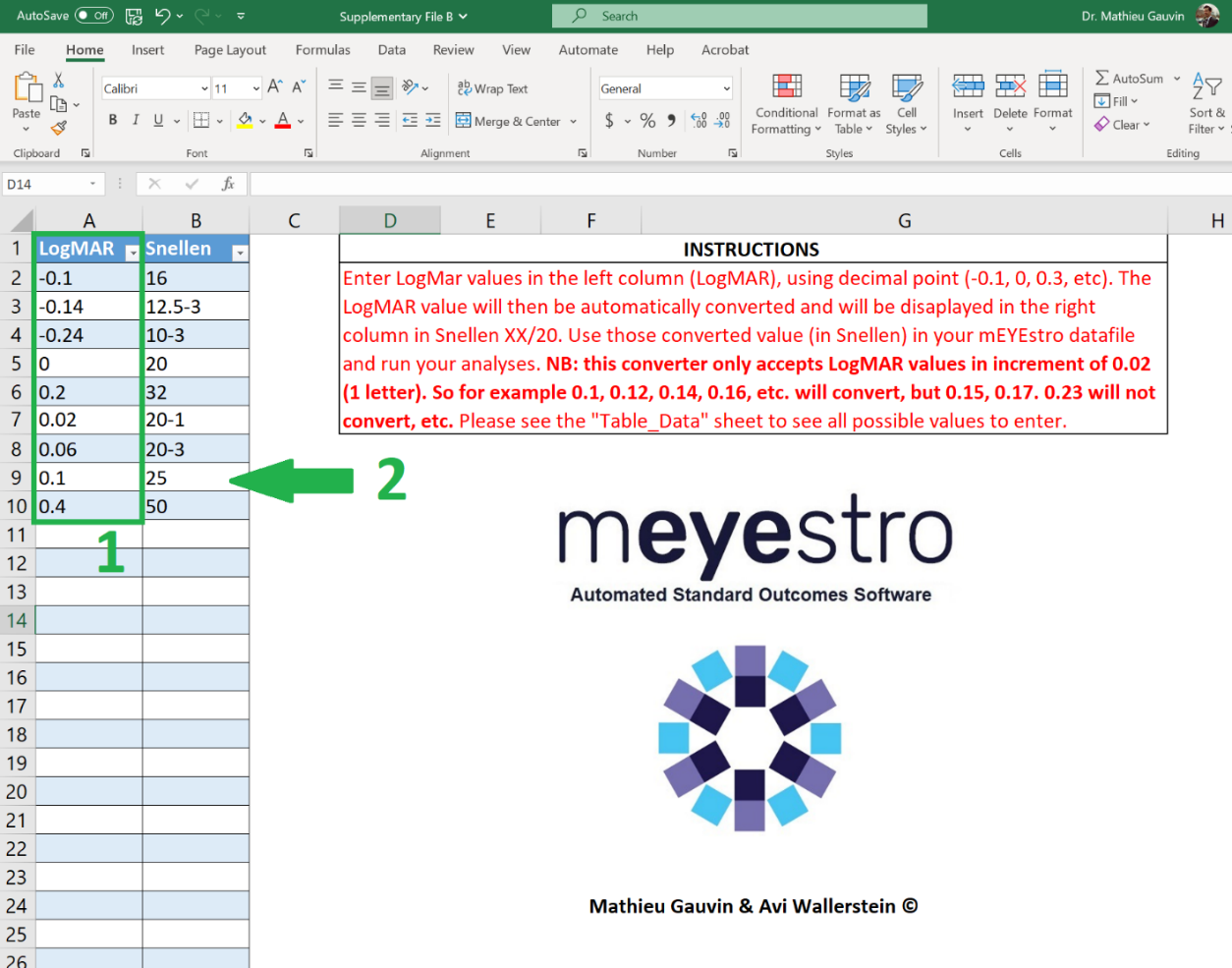


# **What to do if you experiment a bug or crash while using meyestro**

meyestro is a free software under constant improvements and optimizations. If you experience any problems with display and esthetic of the graphs or if meyestro does not respond or crashes, please get in touch with us at [mgauvin@lasikmd.com](mailto:mgauvin@lasikmd.com). Before contacting us, please ensure that your datafile respects the meyestro input format, and that you selected the right options for your dataset. For example, if you chose a “Paired Group” design but your two data files do not have the same number of eyes, you may experience issues with meyestro because a “Paired Design” assumes that the same eyes are being compared, so if you do not have the same number of lines in both data files, meyestro will not work properly.

# **Revision notes**

## **Version 1.8**

1. We added the display of preoperative and postoperative UDVA and CDVA in logMAR values at the top of Panel A.
2. We added an Excel spreadsheet that allow users to conver LogMar values to Snellen denominator. See above section **Optional LogMAR to Snellen denominator (20/XX) conversion** for details.
3. We reprogrammed mEYEstro to be able to accept refraction data at any vertex distances. We therefore added 3 new columns in the mEYEstro Excel data files to allow the user to enter the data at any vertex distance.
